# Ruddlesden–Popper Defects Act as a Free Surface: Role in Formation and Photophysical Properties of CsPbI_3_

**DOI:** 10.1002/adma.202501788

**Published:** 2025-06-16

**Authors:** Weilun Li, Qimu Yuan, Yinan Chen, Joshua R. S. Lilly, Marina R. Filip, Laura M. Herz, Michael B. Johnston, Joanne Etheridge

**Affiliations:** ^1^ School of Physics and Astronomy Monash University Melbourne VIC 3800 Australia; ^2^ Department of Physics University of Oxford Clarendon Laboratory Parks Road Oxford OX1 3PU UK; ^3^ Institute for Advanced Study Technical University of Munich Lichtenbergstrasse 2a D‐85748 Garching Germany; ^4^ Monash Centre for Electron Microscopy Monash University Melbourne VIC 3800 Australia; ^5^ Department of Materials Science and Engineering Monash University Melbourne VIC 3800 Australia

**Keywords:** defects, electron microscopy, halide perovskites, solar cells, structure‐property relationships, vapour deposition

## Abstract

The perovskite semiconductor, CsPbI_3_, holds excellent promise for solar cell applications due to its suitable bandgap. However, achieving phase‐stable CsPbI_3_ solar cells with high power conversion efficiency remains a major challenge. Ruddlesden–Popper (RP) defects have been identified in a range of perovskite semiconductors, including CsPbI_3_. However, there is limited understanding as to why they form or their impact on stability and photophysical properties. Here, the prevalence of RP defects is increased with increased Cs‐excess in vapor‐deposited CsPbI_3_ thin films while  superior structural stability but inferior photophysical properties are observed. Significantly, using electron microscopy, it is found that the atomic positions at the planar defect are comparable to those of a free surface, revealing their role in phase stabilization. Density functional theory (DFT) calculations reveal the RP planes are electronically benign, however, antisites observed at RP turning points are likely to be malign. Therefore it is proposed that increasing RP planes while reducing RP turning points offers a breakthrough for improving both phase stability and photophysical performance. The formation mechanism revealed here can apply more generally to RP structures in other perovskite systems.

## Graphical abstract

This work investigates Ruddlesden–Popper (RP) defects in vapor‐deposited CsPbI_3_ thin films using electron microscopy, density functional theory, and optoelectronic measurements. The atomic positions at the planar defect are found comparable to those of a free surface, revealing their role in phase stabilization. While RP planes are electronically benign, antisites observed at RP corners are likely to be malign.

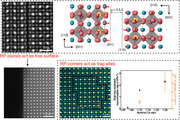

## 1 Introduction

In the past two decades, metal halide perovskite semiconductors have generated considerable attention for their potential application in solar cells.^[^ ^1^, ^2^, ^3^, ^4^ ^]^ Presently, the majority of high‐efficiency perovskite solar cells utilize organic cations, primarily methylammonium (MA^+^) and formamidinium (FA^+^). However, the volatility of these organic molecules promotes material degradation and requires complex solvent‐engineering, presenting a significant obstacle to the long‐term viability of organic–inorganic hybrid perovskite solar cells.^[^ ^5^, ^6^ ^]^ In recent years, all‐inorganic CsPbI_3_ perovskite solar cells have emerged as a subject of intense interest owing to their suitable bandgap and enhanced environmental stability relative to the organic–inorganic hybrid systems.^[^ ^7^, ^8^, ^9^ ^]^ However, the perovskite phases (α, β, and γ) are metastable and quickly transform into a thermodynamically favored non‐perovskite δ phase, which is not photoactive and hence is not suitable for solar cells.^[^ ^10^ ^]^ While various strategies have been explored to prepare CsPbI_3_ solar cells with both high efficiency and structural stability, such as composition engineering, interfacial passivation, and template methods,^[^ ^11^, ^12^ ^]^ it remains a major challenge. In the meantime, vapor deposition is a technique well‐suited for the conformal and homogenous coating of metal halide perovskites, whilst exhibiting excellent compatibility with existing industrial production lines for commercial upscaling.^[^ ^13^, ^14^ ^]^ This solvent‐free method has also allowed for the direct formation of meta‐stable γ‐phase CsPbI_3_ with a low processing temperature.^[^ ^15^, ^16^, ^17^, ^18^, ^19^, ^20^ ^]^

Ruddlesden–Popper (RP) phases are derivatives of the perovskite structure, possessing n‐layers of ABX_3_ interleaved with, typically, AX rock‐salt layers to give a general formula of A_n+1_B_n_X_3n+1_.^[^ ^21^, ^22^ ^]^ RP phases have been reported across a wide variety of halide perovskites.^[^ ^23^, ^24^, ^25^ ^]^ Some are intentionally produced by incorporating large cations, including molecules, as spacers,^[^ ^26^ ^]^ between one or more layers of the perovskite structure (one‐layer corresponding to the so‐called “2D perovskite”, see Figure S2, Supporting Information). Others arise as planar defects where an individual layer of AX lies within an extended matrix of the perovskite structure ABX_3_, so the local composition at the defect plane corresponds to A_2_BX_4_,^[^ ^27^ ^]^ often known as RP defects.^[^ ^25^, ^28^, ^29^ ^]^ RP defects have been proposed to influence the performance of perovskite solar cells through various mechanisms, including promoting ion segregation in mixed‐halide systems,^[^ ^29^, ^30^ ^]^ inducing photoluminescence quenching^[^ ^29^, ^31^ ^]^ and causing quantum confinement effects.^[^ ^23^, ^32^ ^]^ Density functional theory (DFT) calculations suggest that RP defects repel both electrons and holes,^[^ ^33^ ^]^ potentially accelerating rapid degradation by serving as fast ion migration channels.^[^ ^28^ ^]^ However, their role in carrier recombination remains debated: some studies propose that RP defects introduce trap states that lead to non‐radiative recombination,^[^ ^25^ ^]^ while others suggest they enhance radiative recombination through strong charge confinement.^[^ ^32^ ^]^ Despite numerous reports, the detailed atomic‐scale structure of RP defects and their role in the stability and optoelectronic properties of perovskite solar cells are not yet clear.

In this study, we control the prevalence of RP defects by manipulating the nominal ratios between CsI and PbI_2_ in vapor co‐deposited CsPbI_3_ films and determine their effect on the corresponding phase stability and optoelectronic properties. We observe that an excess of Cs is necessary for stabilizing CsPbI_3_ in its photoactive γ perovskite phase and that Cs‐rich compositions promote the formation of RP planar defects which have a locally Cs‐rich structure.

We examine the impact on structure by using quantitative transmission electron microscopy to measure the atomic positions and associated strain at the RP plane. Remarkably, we find the RP plane is comparable in structure to a free surface. This, together with measurements of a related 90° rotation boundary, allows us to identify the mechanism that drives the formation of RP defects and thereby understand how they achieve structural phase‐stability in CsPbI_3_.

We also examine the impact on photophysical properties by measuring the prevalence of RP defects and correlating them with the measured photophysical properties. We use our measurements of the atomic structure and composition of the RP planes and RP turning points to understand their respective impact on electronic structure. We compare DFT calculations of the measured RP planar defect structure with the defect‐free structure. Using these structural measurements and calculations, we reveal the critical and different role of RP planes versus RP turning points in photophysical properties.

Together, these insights open a path for stabilizing the γ‐CsPbI_3_ perovskite phase while optimizing photophysical performance via RP defect engineering.

## 2 Results

### 2.1 Crystallographic Phase and Microstructure Versus Cs:Pb Ratio

We first characterized the microstructure of dual‐source vapor‐deposited CsPbI_3_ films using X‐ray diffraction (XRD) and low‐dose, 4D scanning transmission electron microscopy (4D‐STEM) (see Methodology in Note S1, Supporting Information). The nominal Cs:Pb ratio was varied from 0.85 to 1.25, denoted as Cs‐0.85, Cs‐1.0, Cs‐1.1, and Cs‐1.25 for brevity.

The XRD data was obtained immediately after fabrication and is consistent with the formation of CsPbI_3_ in an orthorhombic γ phase (space group: Pbnm) across all compositions, as shown in Figure S3A–D (Supporting Information). The relative intensity of the perovskite diffraction peak intensities (1st order 002 and 110 pairs, and 2nd order 004 and 220 pairs) changes with Cs composition, with the 001 peak intensity decreasing and the 110 and 220 increasing with increasing Cs, such that for Cs‐1.25 the 004 peak has almost disappeared. This is consistent with a transition from a random orientation to a preferred out‐of‐plane [$1\bar{1}0$] texture, with the growth parallel to the (001) plane suppressed.^[^ ^34^ ^]^ The Cs‐1.25 films also show a small peak corresponding to a secondary phase of CsI, likely due to the high excess of CsI precursor, some of which may not have reacted. (It is noted that while the nominal atomic ratio of CsI:PbI_2_ precursors dictates the shown ratio, the local composition in films may exhibit slight deviations.)

The thin TEM films were examined ≈5 days after fabrication (stored until then in dry nitrogen and darkness). The improved stability with Cs‐excess was observed in the thermal stability (85 °C, N_2_ atmosphere, dark) measurements of as‐prepared γ‐CsPbI_3_ thin films, which show extremely poor stability for stoichiometric Cs‐1.0 and significantly improved stability for Cs‐1.25 (Figure S3E,F, Supporting Information). Cs‐1.0 film exhibited rather poor thermal stability, undergoing a phase change in less than 24 h. In comparison, Cs‐1.25 demonstrated much‐improved durability under heat stress. Interestingly, we observed the disappearance of the excess CsI peak after 48 h, whilst XRD patterns demonstrate improved crystallinity, suggesting further mixing and reaction in the co‐deposited film. Cs‐1.25 film exhibits only a 004 diffraction peak after 144 h. Using 4D‐STEM, the γ‐CsPbI_3_ phase was detected only in the Cs‐rich thin TEM films, with Pb‐rich films Cs‐0.85 and stoichiometric Cs‐1.0 displaying a δ‐CsPbI_3_ phase (space group: Pmnb), as shown in Figure S3G,H (Supporting Information). This γ‐to‐δ phase transition is expected over time due to the known instability of the γ‐phase for stoichiometric or Pb‐rich CsPbI_3_.

Increased grain alignment with Cs‐excess was further confirmed by 4D‐STEM in Figure S3G,H (Supporting Information). Excess Cs also promotes the formation of grains with a more uniform size and shape, as shown in Figure S3G (Supporting Information). As Cs is increased, more of the grains are oriented at or close to the [$1\bar{1}0$] zone axis. Representative diffraction patterns selected from the data sets of the Cs‐1.1 and Cs‐1.25 films are shown in Figure S3H (Supporting Information) and are consistent with γ‐CsPbI_3_ close to the [$1\bar{1}0$] zone axis.

**Figure 1:**
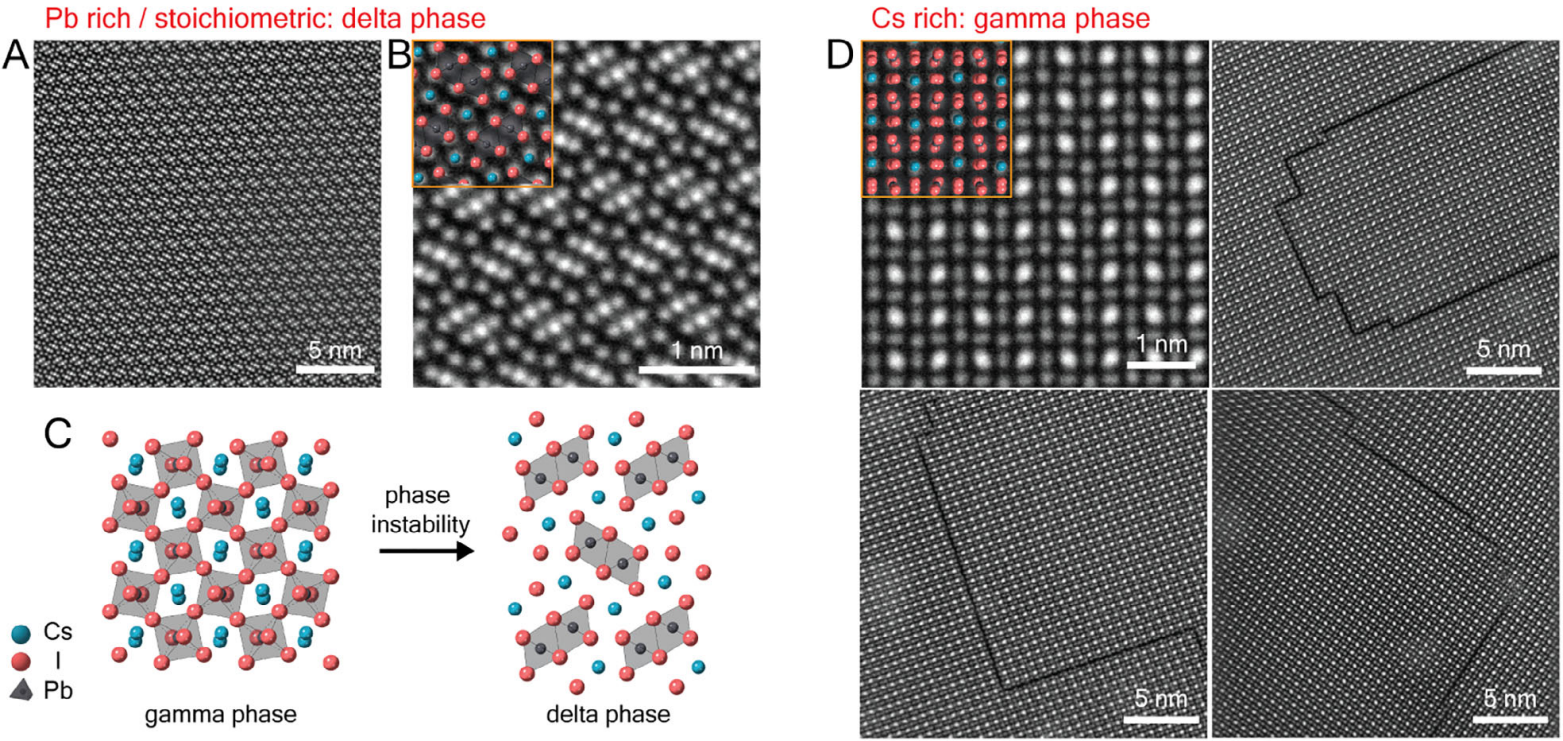
Atomic resolution TEM images of CsPbI_3_ films with different Cs compositions. A,B) STEM‐ADF images of the δ‐CsPbI_3_ phase in Cs‐0.85 films in the [100] zone axis. C) A schematic diagram of the phase transition from the initial γ‐CsPbI_3_ into δ‐CsPbI_3_. γ‐CsPbI_3_ is viewed in the [001] zone axis (Pbnm). δ‐CsPbI_3_ is viewed in the [100] zone axis (Pmnb). D) STEM‐ADF images of the γ‐CsPbI_3_ (top left) and RP defects in Cs‐rich specimens, Cs‐1.1 and Cs‐1.25, viewed in the [$1\bar{1}0$] zone axis. Note, that the highest intensity maxima in the atomic‐number sensitive STEM‐ADF images correspond to columns containing Pb/I atoms (alternating in the beam direction), while the lower and comparable intensity maxima correspond to pure I and pure Cs atom columns.

To investigate further the phase and atomic structure of CsPbI_3_ films, safe dose (5.7 × 10^3^ e A^−2^), atomic‐resolution STEM‐ADF experiments were performed. STEM‐ADF images in **Figure** **1**A,B confirm the Cs‐0.85 and Cs‐1.0 TEM films have the non‐perovskite δ‐CsPbI_3_ phase.

The orthorhombic perovskite γ‐CsPbI_3_ phase is observed in the Cs‐rich TEM specimens in Figure 1D, Cs‐1.1 and Cs‐1.25, consistent with the XRD data. In addition, planar defects parallel to both the (002) and (110) planes are common. As we will demonstrate later, these planar defects have an atomic structure consistent with the RP phase.^[^ ^27^ ^]^ These defects often include steps and 90° turns and, in some instances, form a loop inside the grain. For clarity, hereon, we will call the planar component of the defect, “RP planes”, the turning points “RP TPs” and one‐ or ‐two‐unit cell steps, “RP steps”.

It is important to note that halide perovskites are extremely sensitive to electron irradiation.^[^ ^6^, ^35^, ^36^, ^37^ ^]^ Comprehensive investigations of the structure integrity of RP defects have been conducted and discussed in Note S4 (Supporting Information). The electron dose used throughout this study is well below the beam damage threshold to be sure that the defect and its atomic configuration are intrinsic to the material and have not been created or modified by the electron beam.

### 2.2 Atomic Structure of RP Planes: 90° Boundary, Cs‐Displacements and Octahedral Tilt Relaxation

Two types of RP planar defect are observed, which we will call Type‐90 and Type‐0 (relating to the presence or absence of a 90° rotation boundary, respectively). Both defects can be interpreted as the insertion of an additional Cs plane or the removal of a Pb plane, resulting in a locally Cs‐rich composition. We measure their detailed atomic configuration here, with the key features summarized at the end of this section.

**Figure 2:**
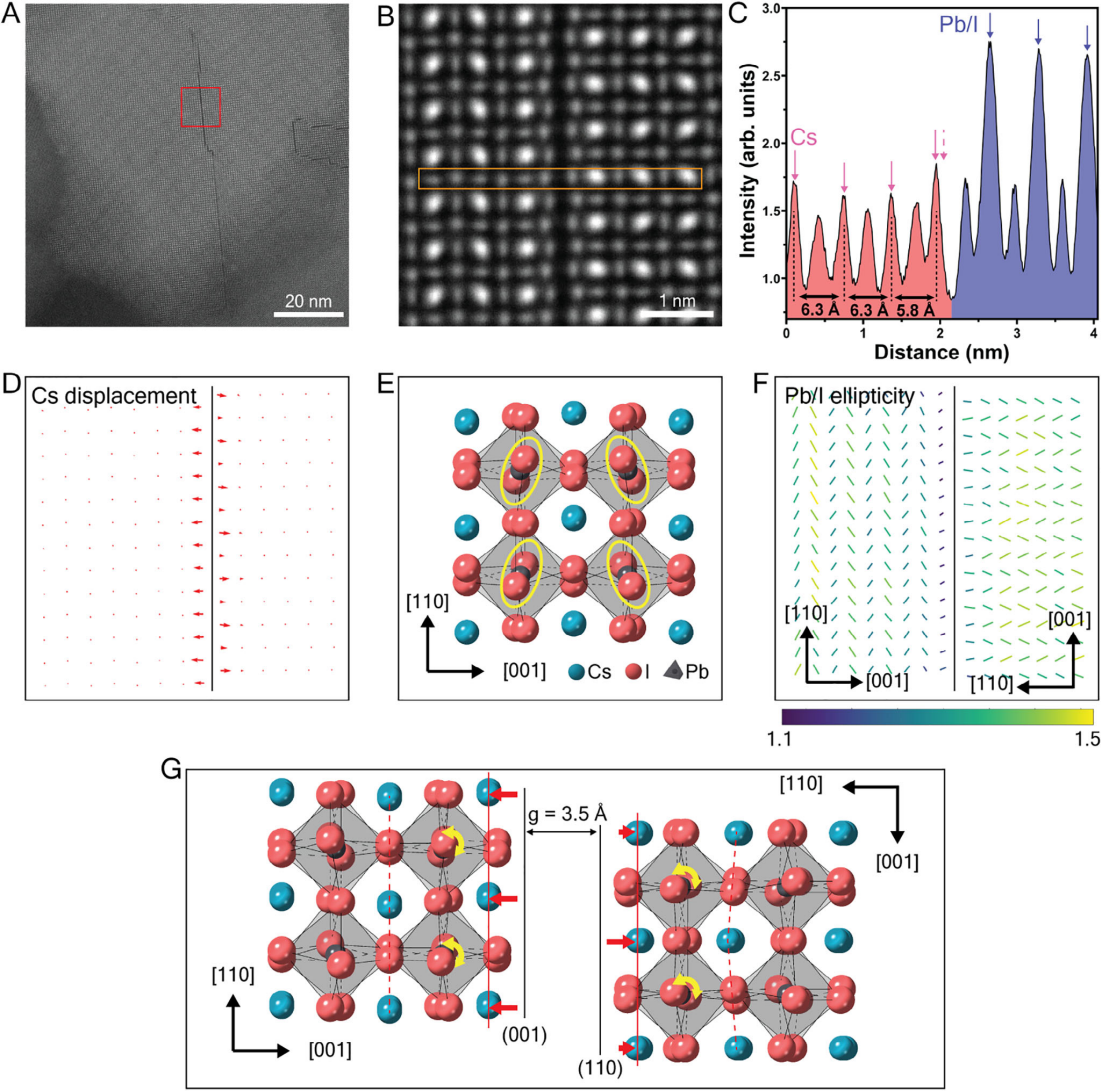
Quantitative analysis of the atomic structure at and around the Type‐90 RP planar defect. A) STEM‐ADF image of a representative grain oriented in the [$1\bar{1}0$] zone axis in Cs‐1.1 film. B) Atomic‐resolution STEM‐ADF image of the RP defect from the region marked in (A). C) Intensity line profile measured from the rectangular region highlighted in (B). Intensity is integrated across the width of the rectangle to enhance signal. D) Cs displacement vector map calculated from measurements of Cs–Cs column distance in (B) revealing shifts of Cs atoms. E) Atomic model of γ‐CsPbI_3_ in the [$1\bar{1}0$] zone axis. Yellow ovals indicate the projected shape of Pb/I columns in the tilted octahedra. F) Ellipticity vector map measured from Pb/I columns in (B) revealing relaxation of octahedral tilt. Line direction indicates the orientation of the ellipse major axis. Line length and color indicate the magnitude of ellipticity (major axis/minor axis). G) Measured atomic model of the Type‐90 RP planar defect showing the gap, Cs displacement, octahedral relaxation, and [001]/[110] axis switch.

The structure of the Type‐90 defect is examined first in **Figure** **2**. It is evident that the defect creates a large gap between two distinct crystal domains, one shifted by half a unit cell relative to the other in the direction parallel to the defect line, resulting in a transition from Pb/I columns to Cs columns across the defect. Furthermore, in ≈20% of defects, there is an in‐plane switch of the crystal axis from [001] (left domain) to [110] (right domain), identified from Figure 2B and from the Fourier transform of the STEM‐ADF images in Figures S6,S7 (Supporting Information). Thus, the Type‐90 RP plane acts as a 90° domain boundary, as illustrated in Figure 2G. Remarkably, given the switch of in‐plane axes across the RP gap, the crystallographic plane is necessarily different on each side of the gap, namely, (001) on one side and (110) on the other (as shown in Figure 2G).

We measured the atomic positions adjacent to the Type‐90 RP plane (from Figure 2C and electron scattering calculations in Note S6, Supporting Information). We find that the Cs columns closest to the RP plane are displaced significantly toward the interior of the domain, in the direction perpendicular to the RP plane, reducing the Cs–Cs column distance relative to the bulk. Figure 2D illustrates the displacement vectors of Cs columns in the vicinity of the RP plane. In the left‐hand domain with the (001) boundary, the Cs columns are displaced by the same amount, so the entire first (001) layer of Cs atoms adjacent to the gap moves collectively inward toward the interior. In the righthand domain with the (110) boundary, the magnitude of the displacement of the Cs columns adjacent to the gap *alternates*. Notably, this results in the first (110) layer of Cs atoms adjacent to the gap being located on exactly the *same* (110) plane (red line in Figure 2G), *unlik*e the Cs columns within the domain interior which lie on alternate (110) planes positioned either side of the corner of the octahedra, as shown by the dotted and dashed red lines in Figure 2G.

Given the observed Cs displacements on each side of the RP gap, we now consider whether there is any accompanying change in the octahedral tilting near the gap. The orthorhombic structure of γ‐CsPbI_3_ entails octahedral tilts in all three axes, as illustrated in Figure 2E. The ellipticity map shown in Figure 2F reveals there is a relaxation of the octahedral tilt angle on each side of the RP gap, as seen from the reduced vector length/intensity in the schematic. The ellipticity map also confirms there is an in‐plane switch of crystal axis between [001] and [110], consistent with the 90° crystal rotation across the gap, as described above.

Finally, we measured the exact magnitude of the gap, the Cs displacement, and octahedral tilt, by quantifying STEM‐ADF images against electron scattering calculations (Note S6, Supporting Information). We determined that the Cs–Cs column distance on the (100) RP plane reduces from 6.3 Å in the interior to 5.8 Å at the interface, while on the (110) RP plane the reduction alternates between 0.2 and 0.5 Å. Similarly, the octahedral tilt angle relative to the bulk was observed to reduce by 33%. The gap distance, as defined in Figure 2G, is 3.5 Å.

We also examined the atomic structure of the Type‐0 RP planar defect in Figure S10 (Supporting Information). In this type, there is no rotation of the domain on each side of the RP gap. Otherwise, the Type‐0 planar defect is essentially the same as the Type‐90 RP planar defect, comprising contraction of the Cs–Cs column distance perpendicular to the defect plane, reduction of the octahedral tilt angle, and a comparable gap size.

The key structural features of the RP planar defects determined above are summarized below:

1. An additional Cs‐plane, giving a local composition of Cs_2_PbI_4_.
2. A gap measuring g = 3.5 Å (see Figure 2G).
3. A half‐unit cell shift parallel to the gap.
4. Cs‐displacements up to 0.5 Å toward the domain interiors.
5. Octahedral tilt relaxation in the first layer adjacent to the RP gap.
6. The first layer of Cs atoms lies “flat” on the (100) or (110) planes on each side of the RP gap.
7. In Type‐90 RP, a 90°crystal rotation across the RP‐gap with different planar interfaces.

### 2.3 Atomic Structure of RP Planar Defects in Three‐ Dimensions

**Figure 3:**
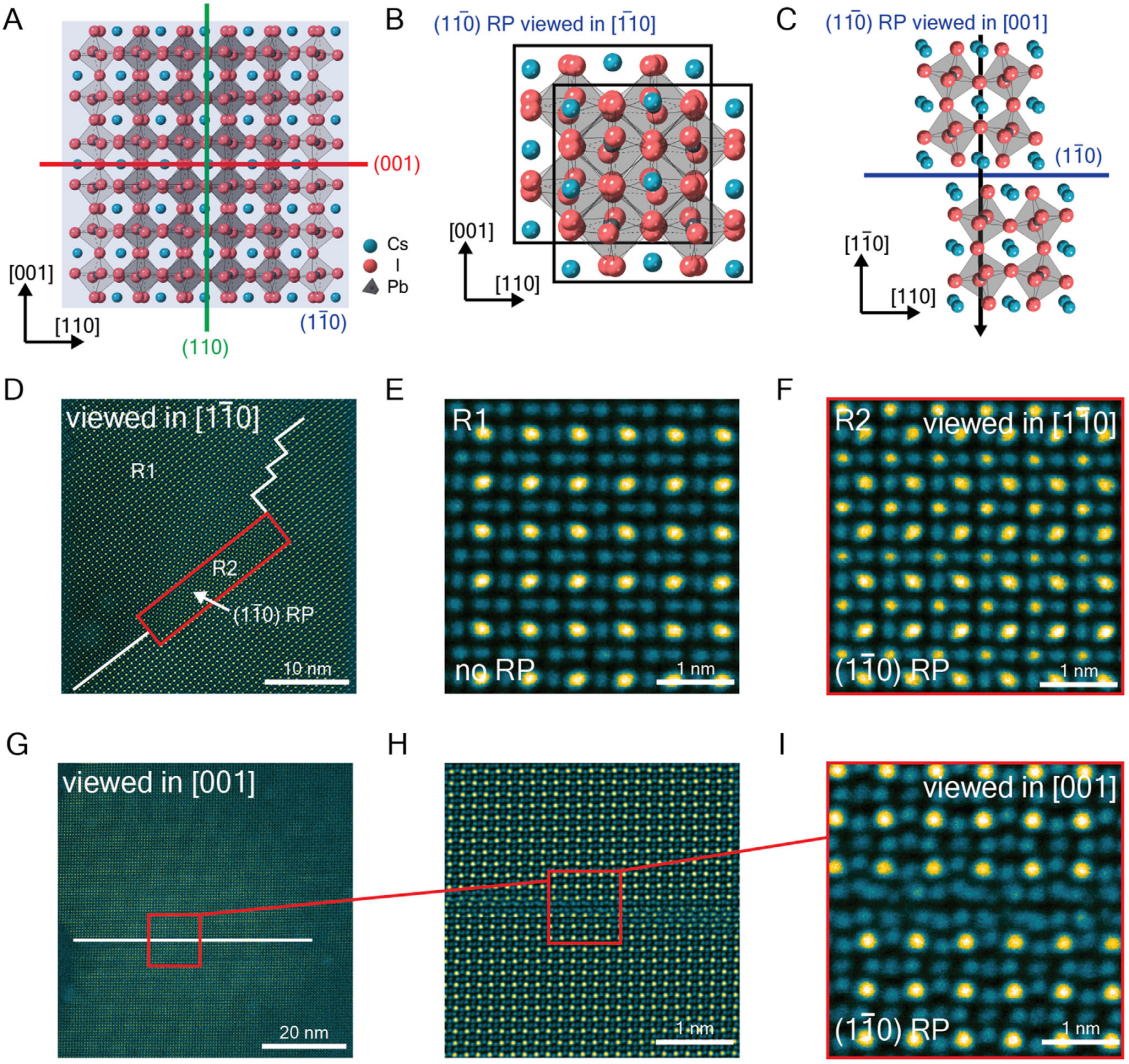
Analysis of ($1\bar{1}0$) RP planes in CsPbI_3_. A) Atomic model incorporating all three orthogonal RP planes viewed in the [$1\bar{1}0$] zone axis. B) Atomic model of ($1\bar{1}0$) RP planes viewed in the [$1\bar{1}0$] zone axis. C) Atomic model of ($1\bar{1}0$) RP planes viewed in the [001] zone axis. D) A representative [$1\bar{1}0$] orientated grain containing ($1\bar{1}0$) RP planes. E) Enlarged region free of RP planes. F) Enlarged region containing ($1\bar{1}0$) RP planes. G) A representative [001] orientated grain containing ($1\bar{1}0$) RP planes. H) Enlarged region from the red box in (G). I) Enlarged region from the red box in (H).

Due to the preferred grain orientation along the [$1\bar{1}0$] zone axis, particularly in Cs‐excess specimens (Figure S3, Supporting Information) where RP defects tend to form, our discussion of RP defects has focused on this projection. In the [$1\bar{1}0$] zone axis, (001) and (110) RP planes can be observed directly, whereas in‐plane ($1\bar{1}0$) RP defects are not easily visible, as shown in **Figure** **3**A. However, ($1\bar{1}0$) RP planes can still be identified from the [$1\bar{1}0$] projection based on the relative intensity of Cs and Pb/I columns. These defects induce lattice shifts of half a unit‐cell along the [110] and [001] directions as illustrated in Figure 3B,C.

Figure 3D shows a representative grain containing ($1\bar{1}0$) RP planes within the region highlighted by the red box. In the defect‐free region (Figure 3E), Pb/I columns exhibit significantly higher intensity than Cs columns. In contrast, within the ($1\bar{1}0$) RP region (Figure 3F), Pb/I columns are shifted beneath Cs columns in the film thickness direction, resulting in a marked increase in the intensity compared with the Cs‐only columns in the defect‐free region.

To provide direct evidence of ($1\bar{1}0$) RP planes, we examined grains oriented along the [001] zone axis (Figure 3G). The observed defects are consistent with the model presented in Figure 3A–C, confirming that RP planar defects exist on three orthogonal planes, with a corresponding release of strain in three dimensions

From atomic‐resolution imaging over 20 grains, we find that ($1\bar{1}0$) RP planes occur less often compared with (001) and (110) RP planes. This suggests that RP defects where the defect plane is perpendicular to the film surface are generated preferentially compared with those parallel to the film surface. This is likely due to the anisotropic strain distribution that arises from the highly anisotropic geometry of film specimens. The film surface acts to reduce strain, so the need for an RP plane (which acts like a free surface) is reduced.

**Figure 4:**
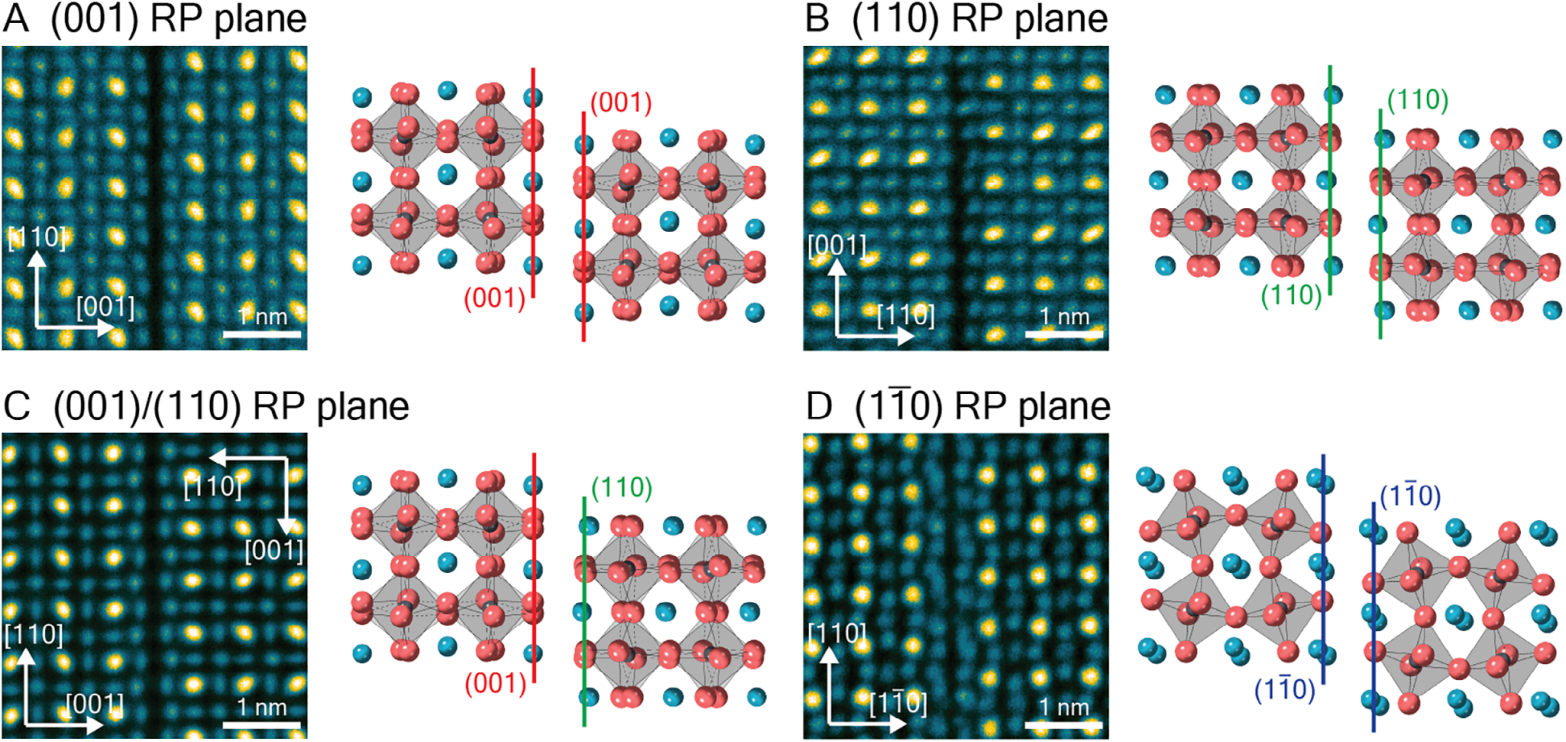
Summary of different types of RP planar defects in CsPbI_3_. A) (001) type‐0 RP defect. B) (110) type‐0 RP defect. C) (001)/(110) type‐90 RP defect. D) ($1\bar{1}0$) type‐0 RP defect.

A summary of the different RP defect types observed in this work is provided in **Figure** **4**. Three orthogonal RP planes are found and are connected in three dimensions through corner turning. In the definition of the orthorhombic unit cell, these RP planes correspond to the (001), (110), and ($1\bar{1}0$) planes.

### 2.4 Atomic Structure of RP Turning Points: Antisites and Dangling Bonds

**Figure 5:**
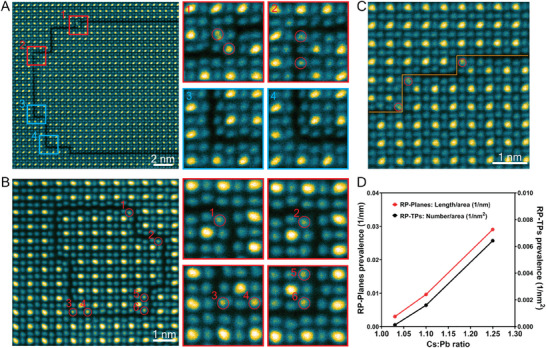
A–C) Antisite defects at RP turning points in Cs‐1.1 films; STEM‐ADF images showing the Pb_Cs_ antisite defects at turning points highlighted in red (but absent in blue). A) Type‐0 RP defect. B) Type‐0 RP defect forming a loop. C) Type‐90 RP defect. D) Prevalence of RP planes and RP turning points in different Cs:Pb ratio films.

In addition to the RP planes, we frequently observed points at which the RP planes turn and switch direction by 90°. These “turning points” are structurally distinct from the RP planes and may serve as hotspots for atomic defects. We therefore discuss these RP turning points separately in this section. Careful analysis of STEM images reveals that antisite defects at the turning points are common. In the STEM‐ADF image, **Figure** **5**A, the nominal position of the Cs columns at some RP turning points show a significantly higher intensity than for the Cs column positions in the bulk. Given the relative atomic number of Pb and Cs, these higher intensity maxima can be attributed to the presence of Pb in these columns, suggesting the formation of Pb_Cs_ antisite defects.

We examined various manifestations of RP defects, including those originating from grain boundaries (Figure 5A), forming RP loops (Figure 5B), or associated with a 90°crystal rotation (Figure 5C). In all of these cases, the presence of antisite defects at turning points was often observed.

### 2.5 Relationship between Prevalence of RP Defects and Cs:Pb Ratio

We estimated the prevalence of RP planes and RP turning points versus Cs:Pb ratio. To quantify the measurement of prevalence, we used the total area of RP planar defects and the total columnar length of RP turning points within the total volume examined (Figure S11, Supporting Information). Many fields of view (> 20 grains) were analyzed to ensure good statistics. The resulting statistics indicate that the prevalence of both RP planes and RP TPs increases significantly with increasing Cs:Pb ratio (Figure 5D). Given the RP plane is in effect an additional Cs‐plane, giving a local composition of Cs_2_PbI_4_, it is expected that Cs‐excess is associated with increased prevalence of the RP defects.

### 2.6 Formation Mechanisms of RP Defects: The Gap Acts as a Free Surface

To provide insights into the mechanisms driving the formation of RP defects in Cs‐rich CsPbI_3_ films, we consider separately two key features of the RP defect structure: the gap (always present) and the 90° boundary (sometimes present). To do this, we measure two separate structures; a free surface in Cs‐rich CsPbI_3_ film and a 90° boundary *without* a gap nor a half‐unit cell shift in the stoichiometric CsPbBr_3_ (choosing the bromine homologue because of the phase instability of stoichiometric CsPbI_3_).

**Figure 6:**
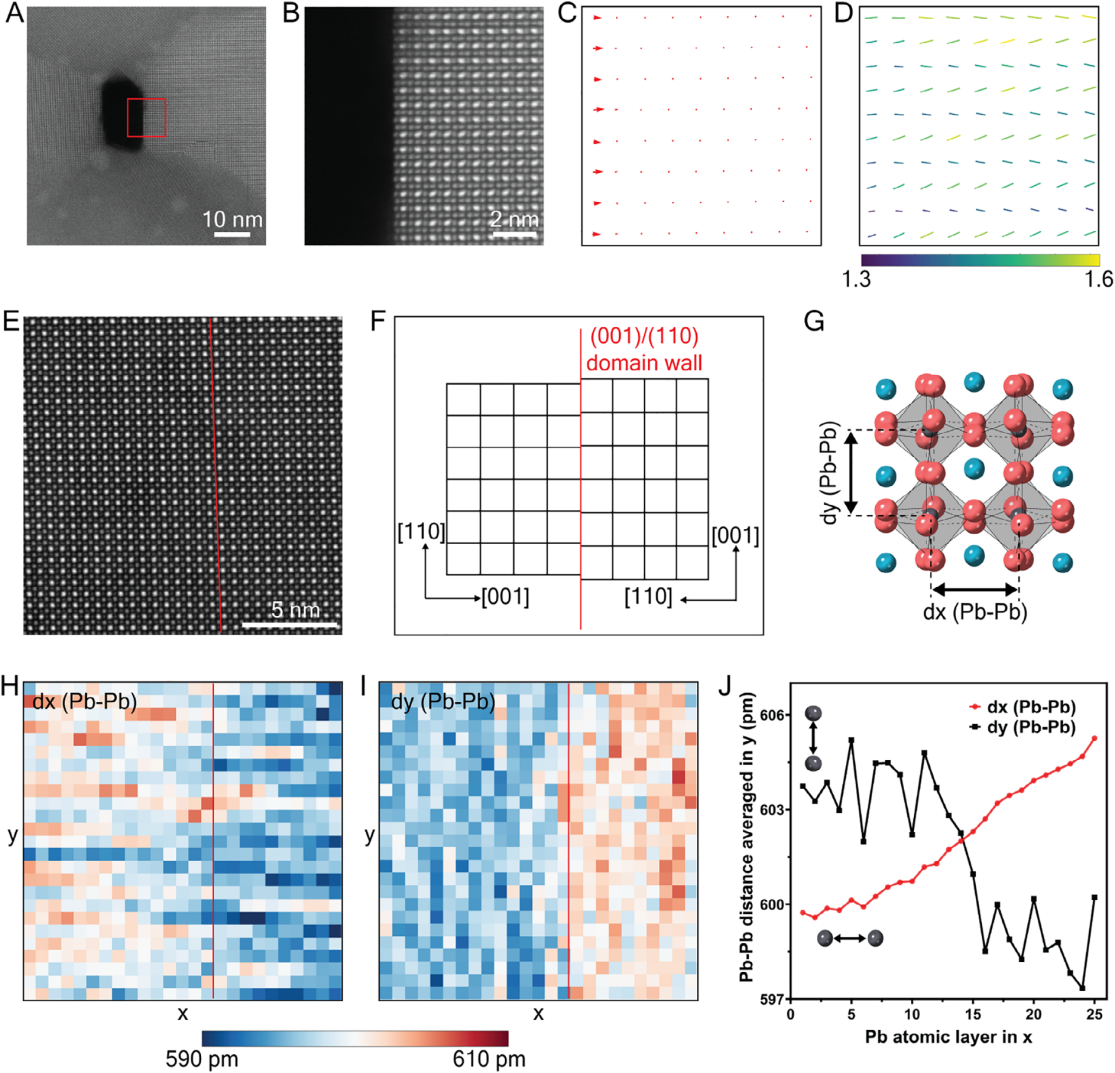
Insights into RP defect formation. Structure relaxation at a surface and strain at gap‐less (001)/(110) boundary A) STEM‐ADF image of a pinhole in the Cs‐1.1 film in the [$1\bar{1}0$] zone axis. B) STEM‐ADF image of the surface region from the red box marked in (A). C) Cs displacement vector map at the surface in (B), comparable to the RP plane. D) Map of the orientation and magnitude of the ellipse major axis measured from the intensity distribution at Pb/I columns in (B) revealing relaxation of octahedral tilt comparable to RP plane. Line direction indicates the orientation of the ellipse major axis. Line length and color indicate the magnitude of ellipticity (major axis / minor axis). E) STEM‐ADF image of CsPbBr_3_ film oriented in the [$1\bar{1}0$] zone axis showing a gap‐less (001)/(110) domain boundary. F) A schematic diagram illustrates the lattice mismatch at the domain boundary. G) Lattice strain near the boundary can be measured from the distance of Pb/I columns (denoted as Pb–Pb distance) in directions parallel (defined as the x direction) and perpendicular (defined as the y direction) to the boundary. H) Pb–Pb distance measured in the x direction and I) y direction. J) Pb–Pb distance measurements in (H,I) averaged in the y direction.

The atomic structure of the CsPbI_3_ film at a free surface, that is, at an interface with the vacuum, was examined in **Figure** **6**A, which shows a rare pinhole in the Cs‐1.1 CsPbI_3_ film. At this surface, Cs displacements and octahedral relaxation are evident, which we measured to be comparable to the RP planar defects, as illustrated in Figure 6B–D. *Remarkably, this suggests that the gap at the RP plane acts as a free surface*. This is a critical observation with implications both for phase stabilization and photophysical properties.

It has been calculated that there is a significant internal strain inherent in orthorhombic γ‐CsPbI_3_ films.^[^ ^38^ ^]^ The observation here that the RP gap acts as a free surface provides an underpinning reason as to why the RP defect stabilizes the γ‐CsPbI_3_ phase in films, namely, by reducing this internal strain, which we discuss later. Moreover, the Cs excess facilitates the formation of the defect by enabling the additional Cs atomic plane, enhancing stability.

### 2.7 RP Gap Relieves Strain at Grain Boundaries: Example, the 90° (110)/(100) Domain Boundary

In the orthorhombic phase of γ‐CsPbI_3_ (and γ‐CsPbBr_3_), the planar distance of (110) planes is different from (001) planes. Consequently, significant lattice mismatch would be expected across a 90° (110)/(100) domain boundary, as illustrated schematically in Figure 6F, inducing significant tensile and sheer strain. We hypothesize that in the presence of excess Cs, the Type‐90 RP planar defect can form, with the gap acting as a free surface, removing this strain.

We explore this hypothesis by measuring the degree of tensile and sheer strain in the *absence* of a gap at a 90° (110)/(001) domain boundary in the stoichiometric, phase‐stable homolog γ‐CsPbBr_3_ films (prepared in the same way as γ‐CsPbI_3_ films, Figure S12, Supporting Information). (Such observations are not possible in phase unstable stoichiometry γ‐CsPbI_3_ films.) This domain boundary is in the field of view in the STEM‐ADF image in Figure 6E but is easily missed without careful analysis and has not been reported previously, to the best of our knowledge.

Unlike the Type‐90 RP planar defect in γ‐CsPbI_3_ films, there is no gap between the two domains and no half‐unit cell shift. The lattice mismatch induces tensile and shear strain in the direction perpendicular and parallel to the boundary. The resultant strain is evident from measurements of the Pb–Pb distance as illustrated in Figure 6F. Figure 6H–J shows a gradual increase in Pb–Pb distance across the boundary in the direction perpendicular to the boundary, together with a sudden decrease at the boundary in the parallel direction. Significantly, there is also no Cs displacement at the domain boundary relative to the interior in Figure S13 (Supporting Information), suggesting this only happens when an RP planar defect is formed, and a free surface is created at the gap.

The observations above are consistent with the hypothesis that the gap at the RP defect plays a significant role in the relief of internal strain associated with incoherent grain boundaries. The specific example of the (110)/(100) 90° domain boundary is a case in point. It forms in the transition from cubic to orthorhombic γ‐CsPbI_3_ when the lattice parameters along the [001] and [110] directions (i.e., lattice parameters b and c in its cubic phase) cease to be equivalent. The tensile strain from the lattice mismatch provides a driving force for the two domains to be separated by a gap, while the shear strain provides a driving force for the half‐unit cell shift parallel to the boundary.

### 2.8 Photophysical Properties Versus Cs:Pb Ratio

**Figure 7:**
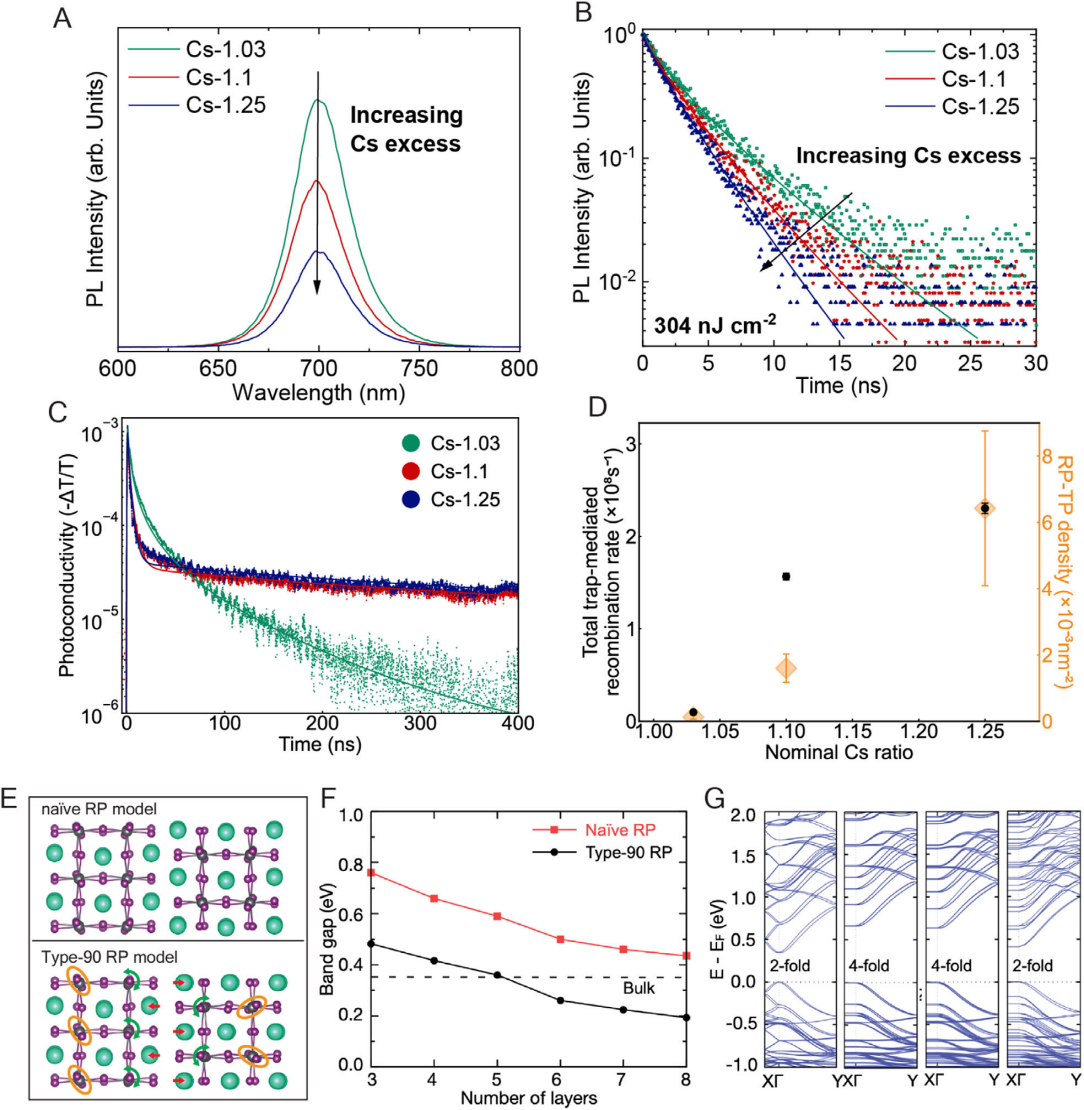
Photo‐physical characterization and density‐function theory simulation of CsPbI_3_. A) Photoluminescence (PL) spectra of 35 nm thick CsPbI_3_ films of various nominal Cs:Pb ratio co‐deposited on z‐cut quartz substrates, following photo‐excitation with a 398 nm‐wavelength continuous wave laser; B) Time‐correlated single photon counting of the same films, after excitation with a 398 nm‐wavelength pulsed diode laser at a repetition frequency of 10 MHz, and fits (solid lines) to transients with a stretched exponential model; C) Photoconductivity transients measured via time‐resolved microwave conductivity (TRMC) for films excited with a fluence of 32 µJcm^−2^ (concurrently acquired time‐resolved PL (TRPL) transients are shown in Figure S18, Supporting Information). Films were prepared as described above (Figure 7A), and all photo‐physical measurements were performed with samples held in an N_2_ atmosphere. TRMC and TRPL transients were simultaneously fitted with a dynamic recombination model, with the results plotted as solid lines; D) The total trap‐mediated recombination rate extracted by fitting the dynamic recombination model to concurrently acquired TRMC and TRPL transients, plotted alongside the prevalence of RP‐turning points as measured via STEM‐ADF, both as a function of the nominal Cs:Pb ratio; E) RP planar defect atomic models; “real” model of Type‐90° defect using measured atomic positions incorporating octahedral relaxation, Cs displacement and 90°crystal rotation, and “naïve model” without octahedral relaxation, Cs displacement (i.e., using same atomic positions as domain interior) nor 90°crystal rotation. F) Calculated DFT bandgaps for real models (black circles) and naïve models (red squares) as a function of the number of octahedral layers in the slab. The bandgap of bulk orthorhombic CsPbI_3_ is shown in a dotted line for comparison. G) Band structures calculated from DFT, including spin–orbit coupling, of a four‐layer defect interface model and an 8 × 2 × 2 CsPbI_3_ supercell. Direction Χ to Γ in reciprocal space corresponds to the direction with the largest lattice parameter in real space (perpendicular to the RP defect), while Γ to Y corresponds to an in‐plane direction in real‐space. From left to right are results on the bulk model, Naïve RP model, Type‐0 RP model, and Type‐90 RP model.

We examined the optoelectronic properties of 35 nm thick vapor‐deposited γ‐CsPbI_3_ films with different nominal Cs:Pb ratios on z‐cut quartz substrates (**Figure** **7**; Figures S14–S19, Supporting Information). Similar measurements of device‐thickness γ‐CsPbI_3_ films and films deposited on TEM grids are shown in Figures S16,S17 (Supporting Information), which illustrate identical trends. As Cs excess is necessary for the stabilization of γ‐CsPbI_3_, studies were performed on the near‐stoichiometric Cs‐1.03 films, together with the more phase‐stable Cs‐1.1 and Cs‐1.25 films. Photoluminescence (PL) spectra in Figures 7A and S14 (Supporting Information) indicate no considerable shift in the emission peak wavelength with compositional variation. Notably, the unnormalized PL peak intensity reduces significantly with increasing Cs‐excess. Similarly, effective lifetimes extracted from time‐correlated single photon counting measurements (TCSPC) in Figures 7B and S15 (Supporting Information) exhibit a decreasing trend with rising Cs concentration. These observations suggest that the use of a more Cs‐rich precursor stoichiometry leads to an increase in the rate of non‐radiative charge‐carrier recombination.

As illustrated in Figures 7C and S18 (Supporting Information), we further probed charge‐carrier recombination dynamics via the concurrent acquisition of time‐resolved photoluminescence (TRPL) and time‐resolved microwave conductivity (TRMC) transients measured under identical optical excitation conditions.^[^ ^39^ ^]^ For Cs‐1.1 and Cs‐1.25 films, a significant remnant photoconductivity component (Figure 7C), is observed with a concomitant elimination of PL signal (Figure S18, Supporting Information). Such a coupled response is indicative of trapping via species‐specific retaining trap states: one charge‐carrier species is preferentially trapped in a retaining trap state, with the other species thus being unable to recombine (the source of the remanent photoconductivity signal).^[^ ^39^ ^]^ We observe a more substantial initial drop in the photoconductivity signal for the Cs‐1.25 sample compared to the Cs‐1.1 sample (Figure S19, Supporting Information), despite a very similar rate of recombination via retaining traps. We attribute this to a 50% increase in the fast‐releasing‐trap‐mediated recombination rate for the Cs‐1.25 sample compared to the Cs‐1.1 sample.

To investigate quantitative differences in recombination behavior between the three films studied, TRMC and TRPL transients were simultaneously fit with a dynamic recombination model (see Note S1, Supporting Information for details). As demonstrated in Figures 7C and S18 (Supporting Information), the model was able to reproduce both the measured photoconductivity and photoluminescence response. Figure 7D shows the total trap‐mediated recombination rate extracted from the model fitting as a function of the nominal Cs:Pb ratio: a monotonic rise is observed in the rate of charge‐carrier recombination via trap states as the Cs‐excess is increased.

Interestingly, there was no discernible change in effective electron‐hole sum charge‐carrier mobility, as determined from TRMC measurements, between the three nominal compositions considered (Table S3, Supporting Information).

In summary, the above results indicate that increasing Cs excess correlates with an increase in the rate of trap‐mediated recombination, suggesting a concomitant rise in the formation of defect states.^[^ ^40^, ^41^ ^]^ Given Cs excess encourages the formation of RP defects, it is pertinent to ask whether RP defects could be acting as trapping sites for photoexcited charge‐carriers in γ‐CsPbI_3_? In the structural observations above, we observe that RP planes act as free surfaces and RP turning points often contain antisites. We thus consider the impact on the electronic structure below using DFT calculations.

### 2.9 Density Functional Theory Calculations of RP Defects

We next performed DFT calculations (including spin–orbit coupling effects; see Supporting Information for computational details) to predict the effects of RP planar defects and their local atomic structures on the electronic band structure. As illustrated in Figures 7E and S20 (Supporting Information), four structural models were built, namely the “bulk model” (perovskite without RP defect); a “naïve model” (perovskite with an incorrect RP model whereby the atomic positions are unchanged from the bulk perovskite); a “Type‐0 RP model” and a “Type‐90 RP model”, with atomic positions as measured using TEM above, incorporating Cs displacement, octahedral relaxation in both models and 90° crystal rotation in the Type‐90 RP model.

To probe the reliability of our atomistic models, we calculate bandgaps for different RP planar defect concentrations by varying the number of octahedral layers (slab thickness) from 3 to 8 for both the Type‐90 RP and naïve RP models. In Figure 7F and Note S12 (Supporting Information), we show that as the slab thickness increases, the calculated bandgap converges toward the bandgap of the bulk, with the gap of the eight‐layer defect model only 0.09 eV away from that of the orthorhombic bulk structure. This is consistent with the expectation that the effect of defect interfaces will be less pronounced with decreasing concentration. We note that all DFT calculated bandgaps are expectedly underestimated with respect to the experiment by more than 1 eV, as documented extensively in the literature.^[^ ^42^, ^43^ ^]^ Furthermore, we find that band edge shapes do not change significantly as the slab thickness changes. These tests confirm that our analysis of the electronic structure for the four‐layer thick structure discussed in the following is physically meaningful and relevant for the description of the optoelectronic properties of CsPbI_3_ thin films inclusive of RP defects.

To investigate further how the defect interface affects the electronic properties, we calculate the band structures of the four‐layer Type‐0 RP, Type‐90 RP, and naïve‐RP models, as well as an 8 × 2 × 2 CsPbI_3_ bulk supercell, shown in Figure 7G.

In the band structure of the bulk CsPbI_3_ supercell, we see more intricate band crossings along X to Γ than Γ to Y, which can be attributed to band folding. Comparing the band structure of the bulk supercell with that of the naïve‐RP model, we observe that the band dispersion along X to Γ is completely suppressed, due to the incorporation of the RP defect (i.e., gap and lattice shift at the RP plane, Table S4, Supporting Information). Furthermore, the VBM (valence band maximum) and CBM (conduction band minimum) in the naïve‐RP model is a 4‐fold degenerate state (including spin–degeneracy), by contrast with the band edges of the bulk structures which exhibit a 2‐fold degeneracy (due to spin only).

We next compare the three RP models. We find that the Cs displacement and octahedral relaxation (naïve RP vs Type‐0 RP) lead to a marginal reduction in the bandgap by less than 50 meV. The 90°crystal rotation across the gap breaks the 4‐fold degeneracy of the VBM and CBM into two sets of bands separated by ≈0.2 eV (Type‐0 RP vs Type‐90 RP), due to a change in the electrostatic potential upon the 90° rotation of the crystal. The bandgap reduction (as depicted in Figure 7G) and the breaking of the 4‐fold degeneracy by the 90° crystal rotation is consistently observed across all RP defect interface systems with different slab thicknesses, confirming that the origin of this effect is due to the subtle structural changes at the RP interface.

Overall, the band structures for the three RP models shown in Figure 7G exhibit very similar band edge shapes and do not appear to introduce any trap states in the bandgap, consistent with previous studies.^[^ ^28^, ^29^, ^33^ ^]^ Therefore, we conclude that these DFT results show no evidence that the RP planar defects and their associated structural variations would have adverse effects on the optoelectronic properties of CsPbI_3_ thin films.

## 3 Discussion

Measuring and understanding the atomic structure of RP defects enables us to elucidate their role in the phase stability and photophysical properties of CsPbI_3_ with varying Cs:Pb ratios.

We first consider the impact on phase stability. TEM results reveal that RP planar defects possess an additional plane of Cs and I ions, resulting in a Cs_2_PbI_4_ local composition. This is consistent with the observations of RP defects in Cs‐rich conditions and the increase in the prevalence of RP defects with increasing Cs excess.^[^ ^30^, ^32^ ^]^ In other words, the introduction of excess Cs promotes the formation of RP defects to accommodate the CsI‐rich non‐stoichiometric composition.

To understand how RP defects might impact phase stability, we consider how the RP planar defect serves to reduce internal strain. During the preparation and crystallization of polycrystalline CsPbI_3_ films, rapid crystal growth at relatively low temperatures promotes the formation of defects such as grain boundaries and other intragrain defects. Due to the small size of Cs ions, CsPbI_3_ goes through a phase transition by tilting the [PbI_6_]^−^ octahedra from α(cubic) to β(tetragonal) and eventually γ(orthorhombic) at room temperature. It has been shown that the spontaneous strain introduced by the lattice distortion during the phase transition is most pronounced in γ‐CsPbI_3_ compared with other halide perovskites, such as CsPbBr_3_, CsPbCl_3_, MAPbI_3,_ and FAPbI_3_.^[^ ^38^ ^]^ Strain originating from both incoherent boundaries and octahedral distortions, contributes to the large internal strain and inherent phase instability of γ‐CsPbI_3_. This corresponds to cases of stoichiometric CsPbI_3_ and Pb‐rich CsPbI_3_, where the as‐prepared γ‐CsPbI_3_ rapidly transforms into undesired δ‐CsPbI_3_.

While the internal strain associated with the phase‐stable bromine homolog, orthorhombic γ‐CsPbBr_3_, are calculated to be less than for phase‐*un*stable stoichiometric γ‐CsPbI_3_,^[^ ^38^ ^]^ our TEM measurements show there is still significant strain associated with the (001)/(110) 90° domain boundaries in γ‐CsPbBr_3_. Such boundaries arise in the tetragonal to orthorhombic phase transition when lattice parameters along the [001] and [110] directions (i.e., lattice parameters b and c in its cubic phase) cease to be equivalent. Remarkably, in Cs‐rich γ‐CsPbI_3_, these domain boundaries are accommodated with no measurable strain as they only exist in association with the RP defect where we have found its gap acts as a free surface.

This boundary provides a specific example of how the formation of RP planar defects separates otherwise strained crystals into sub‐domains, relieving the strain with the introduction of the RP gap which acts like a free surface. RP planar defects typically penetrate through the crystal together with RP turning points and steps, effectively resolving the lattice mismatch between domains in all dimensions through the provision of a free surface. Consequently, Cs‐excess in γ‐CsPbI_3_ suppresses lattice strain and phase instability by promoting RP defect formation.

We have observed a strong correlation between the prevalence of RP defects and an increase in the rate of non‐radiative recombination. To understand whether this is a coincidence or causative, we consider the structural observations and DFT calculations above. The RP planar defect acts as a free surface with minimal related strain. Furthermore, DFT calculations based on the real structure of the RP planar defect suggest that its impact on the electronic structure, while measurable, is likely to be relatively benign with respect to photophysical properties. This is supported by our observation that the effective electron‐hole sum charge‐carrier mobility, determined from TRMC measurements, exhibits no discernible change as the nominal Cs:Pb ratio (and hence RP defect density) is altered.

On the other hand, at the RP turning points, we observed many Pb_Cs_ antisite defects. Pb atoms located at these antisites (i.e., original Cs sites) are likely to have dangling bonds due to the significantly larger distance to halide atoms on the opposite side of the RP defect. Recent DFT calculations have suggested that the formation of Pb_Cs_ antisite defects^[^ ^31^ ^]^ or the presence of dangling Pb bonds associated with halide vacancies could result in deep‐level trap states^[^ ^33^ ^]^ that are likely to be detrimental to photophysical properties. This aligns well with our observation that increasing the prevalence of RP turning points, and hence antisites and dangling bonds, corresponds with a decrease in optoelectronic performance, as shown in Figure 7. Therefore, the excess content of Cs must be optimized to balance the role of RP defects in both stability and optoelectronic properties. While increasing Cs excess beyond. 1.25 may further enhance stability, it is likely to further compromise optoelectronic performance unless defect engineering can effectively mitigate RP corners or anti‐site defects. Moreover, excess CsI can lead to impurity phases such as CsI as observed in the XRD result of the Cs‐1.25 specimen (Figure S3D, Supporting Information), or the commonly found CsI‐rich Cs_4_PbI_6_ phase.

In this work, we investigated the formation of RP defects induced by excess CsI precursor. In contrast, many other studies have intentionally designed structures that have the RP phase using large organic spacer cations.^[^ ^21^, ^44^ ^]^ These large cations effectively passivate defects and improve environmental stability, but their insulating nature compromises charge carrier transport. The resulting alternating organic–inorganic layers exhibit a strong dielectric contrast, which leads to pronounced dielectric confinement and increased exciton binding energies.^[^ ^44^ ^]^ In comparison, RP defects formed by small inorganic Cs^+^ cations are structural defects rather than quantum wells, exhibiting weaker dielectric confinement due to less tightly bound electrons and holes.

We note that the TEM films studied here are necessarily thinner than would be used in a device. However, our fundamental observations regarding the atomic structure of the RP defect, its formation mechanism, the presence of dangling bonds, and its action as a free surface would all be expected to apply in a thicker film.

## 4 Conclusion

The formation of RP planar defects requires an additional Cs plane at the defect interface and is hence promoted with excess Cs. The RP planar defect provides a mechanism to reduce internal strain and stabilize the orthorhombic γ‐CsPbI_3_ phase through the provision of a free internal surface via the RP gap. This mechanism may well apply to the many other perovskite systems that exhibit RP defects, in ferroelectrics, superconductors, and batteries.^[^ ^45^, ^46^, ^47^, ^48^ ^]^ DFT calculations suggest the RP planar defect is likely to be electronically benign with respect to photophysical properties. However, RP turning points, which also increase with Cs‐excess, support antisite defects with Pb dangling bonds leading to deep trap states detrimental to photophysical properties. Collectively, these observations suggest that tuning Cs‐excess and growth parameters to deliberately incorporate RP planar defects without RP turning points in orthorhombic γ‐CsPbI_3_ may ensure phase stability without impacting optoelectronic properties, opening a new avenue for the development of high‐performance solar cells with phase‐stable CsPbI_3_.

## Conflict of Interest

The authors declare no conflict of interest.

## Author Contributions

M.B.J. and J.E. designed the study and supervised the project. W.L. designed and carried out TEM experiments and analyzed data. Q.Y. optimized and prepared specimens and performed XRD, PL, and TRPL measurements. Y.C. and M.R.F. performed DFT calculations. J.R.S.L. and L.M.H. performed in situ TRPL/TRMC measurements and associated modeling. W.L. and J.E. prepared the manuscript. All authors contributed to the discussion of the results and revision of the manuscript.

## Supporting information



Supporting Information

## Data Availability

The data that support the findings of this study are available in the supplementary material of this article.
