# Supplementary material for: Ruddlesden–Popper Defects Act as a Free Surface: Role in Formation and Photophysical Properties of CsPbI3
Source: Adv Mater. 2025 Jun 16;37(34):2501788. doi: 10.1002/adma.202501788 (PMC12392865; doi:10.1002/adma.202501788)
Supplement: Supplementary file 1 — Supporting Information [file ADMA-37-2501788-s001.docx]

Supporting Information for

Ruddlesden–Popper defects act as a free surface: role in formation and photophysical properties of CsPbI_3_

**Authors:** Weilun Li^1^, Qimu Yuan^2^, Yinan Chen^2^, Joshua R. S. Lilly^2^, Marina R. Filip^2^, Laura M. Herz^2,3^, Michael B. Johnston^2^, Joanne Etheridge^1,4,5*^

**Affiliations:**

^1^ School of Physics and Astronomy, Monash University, VIC, 3800, Australia

^2^ Department of Physics, University of Oxford, Clarendon Laboratory, Parks Road, Oxford OX1 3PU, United Kingdom

^3^ Institute for Advanced Study, Technical University of Munich, Lichtenbergstrasse 2a, D-85748 Garching, Germany

^4^ Monash Centre for Electron Microscopy, Monash University, VIC, 3800, Australia

^5^ Department of Materials Science and Engineering, Monash University, VIC, 3800, Australia

This file includes:

Supplementary Note 1 to 12

Supplementary Figures 1 to 20

Supplementary Tables 1 to 5

Supplementary References 1 to 24

Supplementary Note 1 – Materials and Methods

**Thin film sample fabrication**

Z-cut quartz substrates (U) were sonicated for 5 minutes each in fresh Decon-90 solution, de-ionised water, acetone, and isopropyl alcohol sequentially. Prior to deposition, quartz substrates and TEM grids (Agar scientific, Cu-400 mesh) were O_2_-plasma treated for 10 minutes and 0.3 minutes respectively.

For the deposition of inorganic metal halide perovskite thin films, we employed the technique of dual-source co-evaporation in our custom-built evaporation chamber ^[1–3]^. In summary, the chamber was pumped down to a base pressure below 3x10^-6^ mbar for all depositions. The walls of the chamber were maintained at 17 °C and the substrate at 20 °C via two separate chillers. Rates were monitored through quartz crystal microbalances (QCMs) and controlled through a custom-developed software.

Unless otherwise stated, all CsPbI_3_ samples were continuously stored under N_2_ and never exposed to air for all fabrication steps or characterisations. For the sample transfer from Oxford, United Kingdom to Melbourne, Australia, the samples were stored and securely sealed inside a light-proof, stainless-steel transfer unit in N_2_.

**CsPbI_3_**

For the fabrication of CsPbI_3_ thin film samples, PbI_2_ (Alfa-Aeser, 99.998%, ultra-dry metals base) and CsI (Alfa-Aeser, 99.998% metals base) were chosen as precursors. To improve the controllability and reproducibility of the depositions, we utilised four QCM sensors, as illustrated in **Fig. S1A** below. The evaporation of CsI was controlled via the source sensor reading, whilst the PbI_2_ evaporation was controlled via the readings of ‘total substrate rate’ from a sensor located near the substrate. A 2^nd^ sensor near the substrate with a shutter was used for monitoring and providing cross-check, and the shutter of this QCM was only opened prior to the commencement of deposition. The deposition parameters for each precursor are summarised in **Tab. S1** below. Each precursor was tooled with respect to all relevant sensors. To further increase reproducibility, CsI was topped to (1.1 ± 0.1) g for every deposition. Starting from the stoichiometric composition, where Cs:Pb ratio was aimed at nominally 1:1, further changes to the nominal Cs:Pb ratios were achieved by holding the PbI_2_ evaporation rate constant and varying the CsI evaporation rate. Typical sublimation temperature for CsI was between 400 °C and 430 °C, and from 275 °C to 295 °C for PbI_2_ sublimation. Films of a range of different thickness were prepared. As-deposited films were immediately annealed after deposition in N_2_ atmosphere.

| Nominal Cs:Pb Ratios | 0.85:1 | 1:1 | 1.03:1 | 1.1:1 | 1.25:1 |
| --- | --- | --- | --- | --- | --- |
| CsI Rate (Å/s) | 0.16 | 0.19 | 0.20 | 0.21 | 0.24 |
| PbI_2_ Rate (Å/s) | 0.25 | 0.25 | 0.25 | 0.25 | 0.25 |

**Tab. S1.** Set evaporation parameters for depositing CsPbI_3_ with different nominal Cs:Pb ratios, an error of ± 0.005 Å/s is expected.

**CsPbBr_3_**

For the fabrication of CsPbBr_3_ thin film samples, PbBr_2_ (Alfa-Aeser, 99.998%, metals base) and CsBr (Alfa-Aeser, 99.9% metals base) were chosen as precursors. The evaporation rate was controlled via source sensors located next to PbBr_2_ and CsBr crucibles respectively and cross-checked with two substrate sensors. This is illustrated in **Fig. S1B**. Films were prepared such that both a nominal Cs:Pb ratio of 1:1 and a thickness of 35 nm were achieved. CsBr was sublimated at a rate of (0.22 ± 0.005) Å/s and temperature between 390 °C and 420 °C, while PbBr_2_ was at a rate of (0.25 ± 0.005) Å/s and temperature range of 280 °C to 310 °C. As-deposited films were immediately annealed for 1 minute at 170 °C in N_2_ condition.


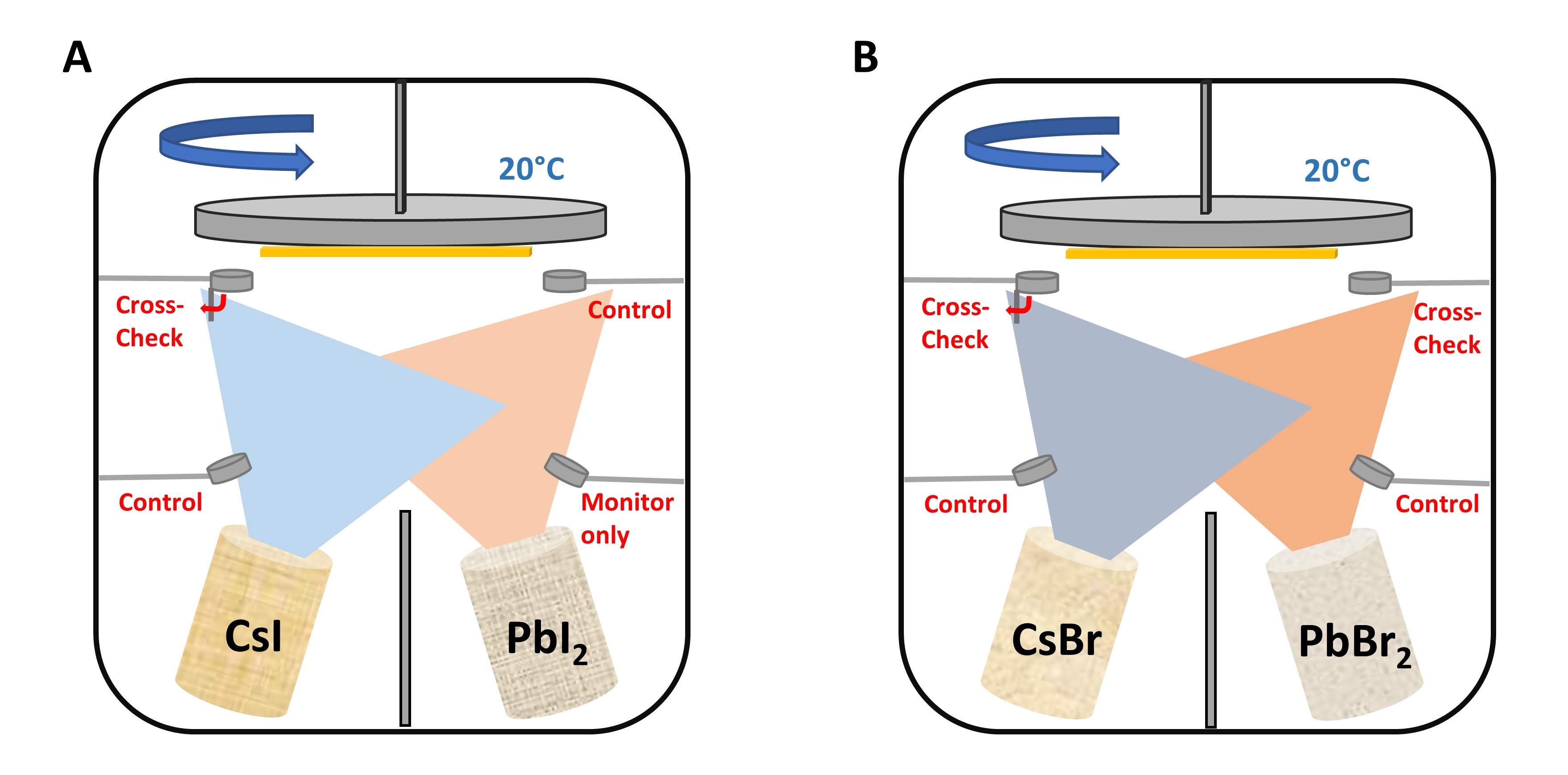


**Fig. S1.** Schematic diagrams of sublimation control, illustrating. (**A)** dual-source co-evaporation for CsPbI_3_; and **(B)** dual-source co-evaporation for CsPbBr_3_.

**Transmission electron microscopy (TEM)**

Thin films suitable for TEM examination were made by direct vapour-deposition onto carbon substrates.

Scanning Transmission Electron Microscope - Annular Dark Field (STEM-ADF) images were taken using a Thermo Fisher Scientific Spectra φ FEG-TEM and a FEI Titan^3^ 80-300 FEG-TEM, both equipped with probe and imaging spherical aberration correctors. Images were acquired at 300 kV, using a 15 mrad probe-forming aperture, and 39-200 mrad detector collection angle. 4D-STEM datasets were collected using an electron microscope pixel array detector (EMPAD), with 256 x 256 probe positions, 128 x 128 pixel diffraction patterns acquired at 1 kHz. 4D-STEM data sets were analysed using an in-house MATLAB code developed by Dr Bryan Esser, Quant4D. Quantitative analyses of the location of intensity maxima in STEM-ADF images were performed using an open-source Python package, Atomap ^[4]^. STEM-ADF images were post-filtered with an Average Background Subtraction Filter to enhance the signal-to-noise ratio. Electron scattering calculations to refine atomic positions from intensity maxima in the image were performed using MuSTEM based on the multislice algorithm ^[5]^. TEM specimens were transferred in a glove box from the light-proof, N_2_-filled transfer unit and mounted on a single tilt holder to the vacuum of the TEM. All TEM experiments followed a “shoot blind” protocol whereby no electron dose was applied to the region-of interest during the experiment set up, neither during zone axis tilting nor parameter tuning.

STEM-ADF image intensity is sensitive to atomic number, so the highest intensity maxima correspond to the atomic columns comprising the highest atomic number. For example, in the [001] zone axis of the γ-CsPbI3 phase (**Fig. 1D**), the highest intensity maxima correspond to alternating Pb and I atoms in the beam direction, while the lower intensity maxima corresponding to neat I and pure Cs atom columns, due to their similar atomic numbers (I=53, Cs=55).

**X-Ray diffraction (XRD) measurement**

XRD patterns were measured with a Panalytical X’pert powder diffractometer with copper X-ray source (Cu-K_α_ 1.54 Å set at 40 kV and 40 mA). All samples measured were deposited on z-cut quartz substrates, and all spectra were further corrected with reference to the z-cut quartz peak at 2θ = 16.4° and 33.1°. During the measurement, samples were held under N_2_ atmosphere through an ‘air-free’ holder.

**Steady-state photoluminescence (PL) measurement**

PL measurements were performed through photoexcitation of perovskite thin films of various thickness deposited on quartz substrates and TEM grids with a 398 nm-wavelength continuous wave laser (PicoHarp, LDH-D-C-405M) with a power density of 3.8 W/cm^2^ from the perovskite side. The emitted PL was coupled into a grating monochromator (Princeton Instruments, SP-2558) and measured with an ICCD camera (Princeton Instruments, PI-MAX4). During the measurement, all samples were held under N_2_ atmosphere through a custom-built sample holder.

**Time-correlated single photon counting (TCSPC) measurement**

TCSPC measurements were carried out through photoexcitation of perovskite thin films of various thickness deposited on quartz substrates and TEM grids with a 398 nm-wavelength pulsed semiconductor diode laser (PicoHarp, LDH-D-C-405M) with a repetition rate of 10 MHz from the perovskite side, with a fluence of either 304 nJ/cm^2^ or 475 nJ/cm^2^. The emitted PL was coupled into a grating monochromator (Princeton Instruments, SP-2558) and collected by a photo-counting detector (PDM series from MPD). Timing was controlled by a PicoHarp300 event timer. During the measurement, all samples were held under N_2_ atmosphere through a custom-built sample holder.

The time-resolved PL transients were fitted to a stretched exponential function in the form of:

$$\begin{aligned} I=I_{0}\exp\left( -\left( \frac{t}{\tau} \right)^{\beta} \right),\#\left( 1 \right) \end{aligned}$$

In which $\beta$ is the stretching parameter. The mean lifetime $\tau_{av}$ is extracted via $\frac{\tau}{\beta}$ , where the PL intensity has dropped by a factor of 1/e.

**Thin film absorption measurement**

Absorption measurements were performed on a Bruker Vertex 80v Fourier Transform Interferometer, with a tungsten-halogen near-infrared source, CaF_2_ beam splitter, and a silicon diode detector. A blank z-cut quartz and a sliver mirror were used as the transmission and reflection reference respectively. All samples measured were co-deposited on quartz substrates.

**Simultaneous time-resolved microwave conductivity (TRMC) and time-resolved photoluminescence (TRPL) spectroscopy**

Experimental details have been previously described ^[6]^. The sample under consideration was photo-excited with pulses from an amplified ultrafast laser system (~ 35 fs pulse duration, 800 nm central wavelength, 5 kHz repetition rate Ti:Sapphire laser system from Spectra Physics: MaiTai – Ascend – Spitfire regenerative amplifier), with the pump frequency being doubled in a BBO crystal (2 mm thick) to a 400 nm central wavelength. A variable ND filter wheel was utilised to control the fluence of the pump. Produced initially via a signal generator, 34 GHz microwave radiation was converted using a frequency tripler to 102 GHz, and subsequently launched into free space via a standard gain feed horn antenna. PTFE lenses were utilised to focus the microwave radiation onto the sample, with the transmitted radiation then re-focussed onto a custom-made GaAs Schottky-diode detector. A broad-band amplifier (fabricated in-house) amplified photo-induced changes in the signal, with the resulting output then being measured with a fast oscilloscope. When measuring the microwave transmission of the sample in the absence of photo-excitation, the broad-band amplifier was removed. A sample of known photoconductivity was utilised to calibrate the TRMC system. Recorded on the same oscilloscope as above and used in conjunction with an in-house fabricated broad-band amplifier, the TRPL response of the sample was measured with a fast photodiode detector (laser scatter was removed by implementation of a spectral filter). The time resolution of the system was previously measured to be 1.1 ns for TRMC transients and 2.6 ns for TRPL transients ^[6]^. For all measurements, the sample was held at room temperature in a nitrogen environment.

**Extraction of trap-mediated recombination rate and effective electron-hole sum charge-carrier mobility** **(dynamic recombination model)**

The total trap-mediated recombination rate was extracted by employing a dynamic recombination model to fit simultaneously acquired TRMC and TRPL data. In this model (see ^[6]^ for more details), charges can recombine though bimolecular recombination (*k*_B_), monomolecularly via non-retaining traps or via phonon-mediated recombination where the capture & the recombination mechanism occur on similar timescales (*k*_M_), or via species-specific retaining-trap states where the capture mechanism (*k*_T_) is much quicker than the escape/recombination mechanism (*k*_E_). The following coupled differential equations model the evolving concentrations of free electrons (*n*), free holes (*h*), and carriers captured in species-specific retaining traps (*n*_T_) with respect to time (*t*):

$$\begin{aligned} \frac{dn(t)}{dt}=-k_{\mathrm{Bi}}n(t)p(t)-k_{M}n(t)-k_{T}n(t);\#\left( 3 \right) \end{aligned}$$

$$\begin{aligned} \frac{dn_{T}(t)}{dt}=k_{T}n(t)-k_{E}n_{T}(t)p(t);\#\left( 4 \right) \end{aligned}$$

$$\begin{aligned} p(t)=n(t)+n_{T}(t).\#\left( 5 \right) \end{aligned}$$

Note, the above notation assumes that retaining traps specifically capture electrons; however, the problem is symmetric, and thus the output of the model can equally describe the case of hole-specific capture.

It has previously been evidenced for lead-halide perovskites that the electron mobility and the hole mobility are very similar ^[7]^. Applying this assumption, the photoconductivity (*σ*) can be defined as ^[6]^

$$\begin{aligned} \sigma\left( t \right)=\frac{\mu e}{2}\left( n(t)+p(t) \right),\#\left( 6 \right) \end{aligned}$$

with *μ* the sum charge-carrier mobility and *e* the elementary charge. The photoconductivity of a thin-film is directly proportional to the photo-induced change in microwave transmission ^[6,8]^. Therefore, the change in microwave transmission of a thin-film caused by the presence of (free) charge carriers should be proportional to the total number of free charge carriers:

$$\begin{aligned} \frac{\Delta T}{T}\left( t \right)\propto\frac{n(t)+p(t)}{2}.\#\left( 7 \right) \end{aligned}$$

Similarly, the photoluminescence intensity should be proportional to the number of charge carriers recombining via radiative (here, assumed to be purely bimolecular in nature) recombination:

$$\begin{aligned} I_{\mathrm{PL}}(t)\propto k_{\mathrm{Bi}}n(t)p(t).\#\left( 8 \right) \end{aligned}$$

Simulating the charge-carrier recombination dynamics as above (Eq. 3 – Eq. 5), corresponding photoconductivity and photoluminescence transients can be modelled (Eq. 7 and Eq. 8 respectively).

Leveraging the mathematical framework outlined above, simultaneously acquired TRMC and TRPL transients measured under the same pulsed excitation conditions were modelled, with optimised fit parameters determined. It was assumed that excited charge carriers were distributed homogeneously, and that the population of free charge-carrier pairs at time zero is equal to the number of incident photons ^[6,9]^. Due to the low repetition rate of the laser used in this experiment, it was further assumed that all trap-states were depopulated at time zero ^[6]^. To account for the non-negligible instrument response time (different for TRMC and TRPL), the transients described by Eq. 7 and Eq. 8 were convoluted with normalised-Gaussian functions. A weighted least-squares algorithm was utilised, where all six transients (TRMC/TRPL for three samples) were simultaneously modelled ^[10]^. Determined optimised recombination coefficients are listed (with a corresponding uncertainty) in **Tab. S2**. *k*_M_, *k*_T_ and the proportionality constants arising from the use of Eq. 7 and Eq. 8 were allowed to vary between the three samples modelled; however, *k*_Bi_, *k*_E_ and the parameters defining the TRMC/TRPL instrument response functions were fit globally across all samples. Errors were estimated using a 50-iteration bootstrap method ^[10]^.

The total trap-mediated recombination rate (see **Fig. 7D**, main text) is the aggregate of both the retaining and non-retaining trap components (i.e., the sum of *k*_M_ and *k*_T_).

From the photoconductivity onset, a value for the effective electron-hole sum charge-carrier mobility (*φμ*) can be calculated ^[8]^,

$$\varphi\mu=-\varepsilon_{0}c\left( n_{A}+n_{B} \right)\frac{A_{\mathrm{eff}}hc}{eE\lambda\left( 1-R_{\mathrm{pump}}-T_{\mathrm{pump}} \right)}\frac{\Delta T}{T}\left( t=0 \right),$$

where *φ* is the photon-to-free-charge-carrier branching ratio, *h* is the Planck constant, *ε*_0_ is the permittivity of free space, *c* is the speed of light, *E* is the energy of the excitation pulse, $\lambda$ is the wavelength of the excitation pulse, *A*_eff_ is the effective area of overlap between the pump and probe beam, *n*_A_ and *n*_B_ are the refractive indices (at the probe frequency) of the substrate and air, and *R*_pump_ and *T*_pump_ are the fraction of pump light reflected and transmitted by the sample (here, both taken to be zero). The relative change in microwave transmission at time zero was directly extracted from the model fitting. Computed values (and associated errors) for *φμ* are listed in **Tab. S3**.

| Nominal Cs: Pb | *k*_Bi_ (× 10^-11^ cm^3^s^-1^) | | *k*_M_ (× 10^8^ s^-1^) | | *k*_T_ (× 10^7^ s^-1^) | | *k*_E_ (× 10^-12^ cm^3^s^-1^) | |
| --- | --- | --- | --- | --- | --- | --- | --- | --- |
|  | Value | Error | Value | Error | Value | Error | Value | Error |
| 1.03:1 | 2.68 | 0.15 | 0.0962 | 0.0016 | 0.0109 | 0.0006 | 2.15 | 0.10 |
| 1.1:1 |  |  | 1.32 | 0.04 | 2.46 | 0.02 |  |  |
| 1.25:1 |  |  | 2.02 | 0.05 | 2.85 | 0.02 |  |  |

**Tab. S2.** Optimised recombination coefficients determined from utilising a dynamic charge-carrier recombination model to concurrently fit simultaneously acquired TRMC and TRPL transients measured under the same pulsed excitation conditions. *k*_Bi_ and *k*_E_ are intrinsic properties of the material and retaining-trap species formed respectively and were therefore treated as global fit parameters ^[6,11]^.

| Nominal Cs: Pb | *φμ* (cm^2^V^-1^s^-1^) | |
| --- | --- | --- |
|  | Value | Error |
| 1.03:1 | 2.2 | 0.2 |
| 1.1:1 | 1.9 | 0.2 |
| 1.25:1 | 2.2 | 0.3 |

**Tab. S3.** The effective electron-hole sum charge-carrier mobility for each of the films considered, as determined from time-resolved microwave conductivity measurements. The above values indicate a lower limit for the electron-hole sum charge-carrier mobility: not all incident photons will be absorbed, some photons will generate excitonically bound charge carriers (*φ*<1), and some charge carriers will recombine in the sub-ns regime (instrument response time) through recombination pathways that are not explicitly modelled.

**Density functional theory calculations**

In this work, we carried out first principles calculations based on Density Functional Theory (DFT) ^[12]^ as implemented in the Quantum Espresso package (version 6.7) ^[13,14]^. We use the PBE parameterisation of the Generalized Gradient Approximation (GGA) of DFT ^[15]^, and employ fully-relativistic PBE norm-conserving pseudopotentials from the Pseudo Dojo repository ^[16,17]^, with the following valence electron configurations: Cs 5s^2^5p^6^6s^1^, Pb 5d^10^6s^2^6p^2^, I 5s^2^5p^5^. We use a kinetic energy cutoff of 50 Ry in all calculations and sample the Brillouin zone using a Γ centred 3×3×1 Monkhorst-Pack grid. We include spin–orbit coupling in all calculations reported throughout in this work. All calculated band gaps reported in this work are underestimated with respect to experiment by more than ~ 1 eV, as expected for this level of theory (and documented extensively in the literature ^[18]^)

Supplementary Note 2 – Ruddlesden–Popper Structure in CsPbI_3_


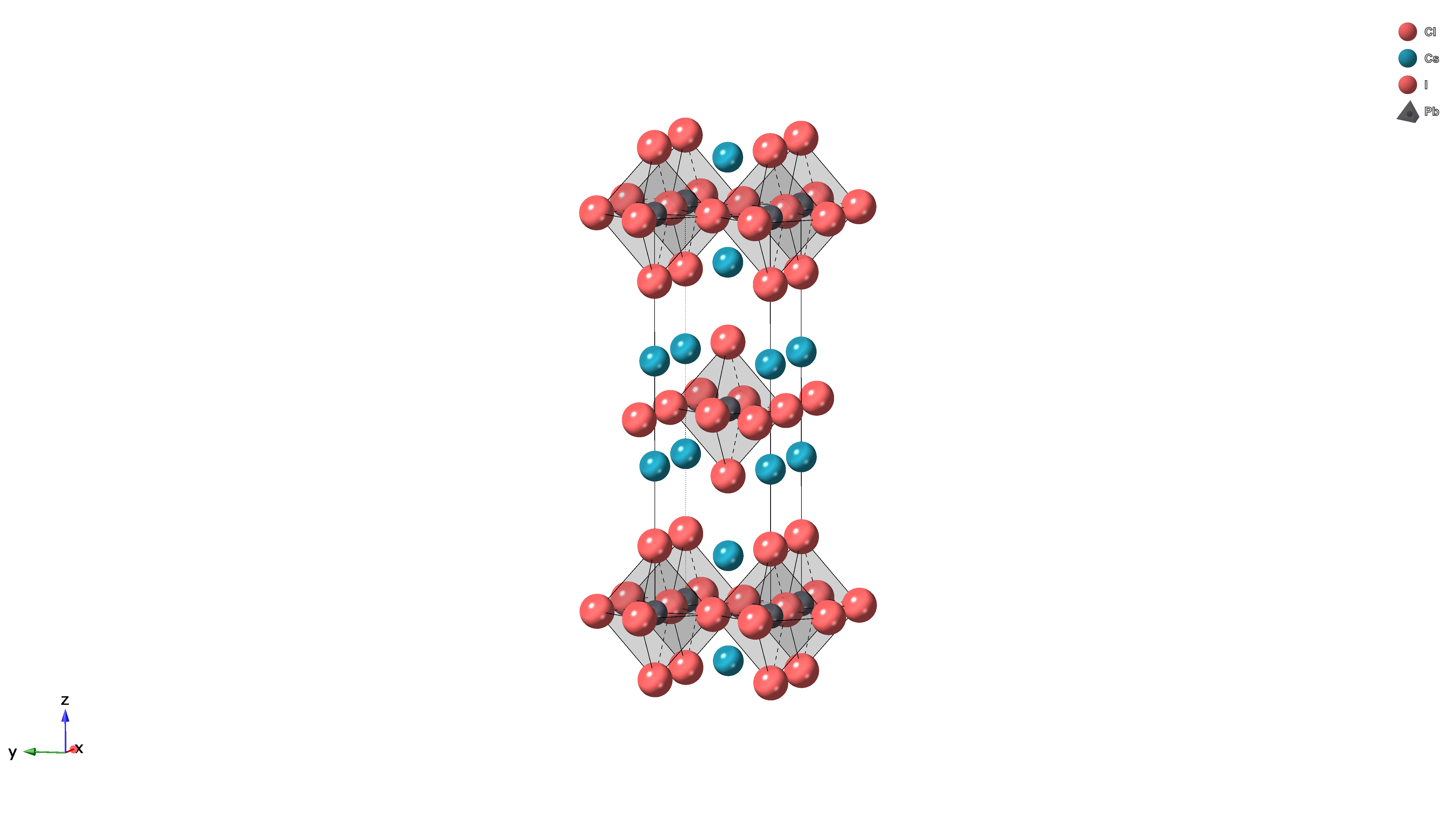


Fig. S2. Crystal model of RP structure in γ-CsPbI_3._

Supplementary Note 3 – Crystallographic Phase and Microstructure versus Cs:Pb Ratio

**
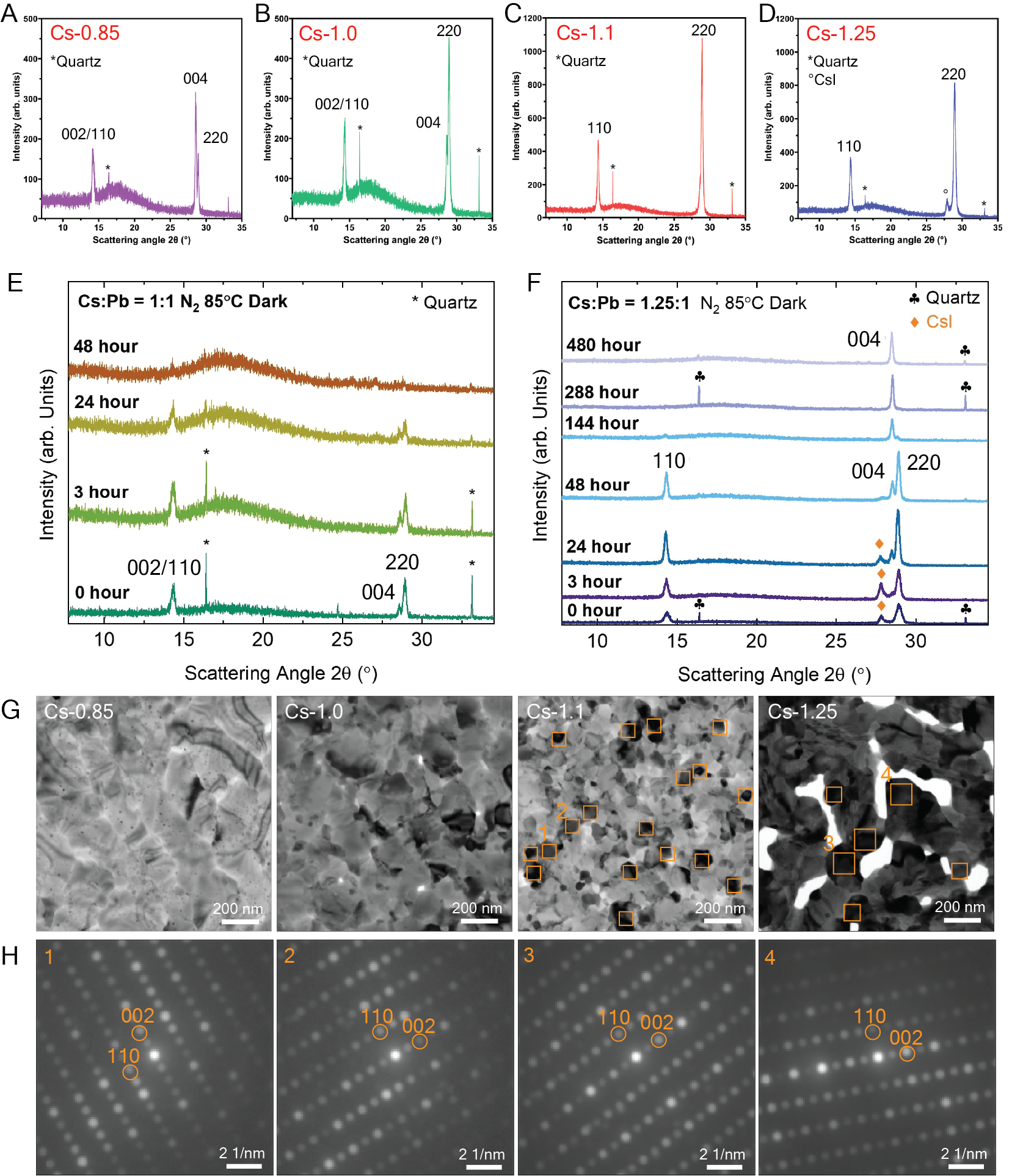
**

**Fig. S3. XRD and 4D-STEM Characterisations of CsPbI_3_ films with varying Cs:Pb ratio. (A)** Cs:Pb = 0.85:1 **(B)** Cs:Pb = 1:1 **(C)** Cs:Pb = 1.1:1 **(D)** Cs:Pb = 1.25:1. A Cu-K_α_ 1.54 Å source is used for XRD. **(E, F)** Thermal stability of co-deposited CsPbI_3_, measured via XRD, illustrating (**E)** Stoichiometric Cs-1.0 films, and **(F)** Cs-1.25 films. Films were placed on a hotplate kept at 85°C in N_2_ atmosphere and kept in dark. **(G)** Bright field images extracted from 4D-STEM data of CsPbI_3_ films deposited directly on TEM grids. Grains marked by orange squares are representative grains oriented close to the [$1\bar{1}0$] zone axis. **(H)** Representative diffraction patterns from the grains in Cs-1.1 and Cs-1.25, as numbered in **(G)**.

Supplementary Note 4 – Material Integrity and Beam Damage

Halide perovskites are well-known to be electron beam sensitive ^[19–22]^. Although all-inorganic halide perovskites have been demonstrated to be much more stable than organic-inorganic hybrid halide perovskites, it is still critical to keep dose to a minimum level that ensures the structural integrity of the RP defects. We investigate what this dose level should be in this Supplementary Note.

Firstly, we note that in the larger field of view images in **Fig. 1D** and **Fig. S4**, a small number of spherical regions, about 2-3 nm in diameter, show higher image intensity. This phenomenon is commonly reported in TEM studies of halide perovskites and has been well studied to be the nucleation of Pb or PbI_2_ nanoparticles ^[23,24]^. Given that this type of beam damage has been discussed extensively elsewhere and does not affect the structure of the RP defects, as shown in **Fig. S4**, it will not be discussed further in this work.


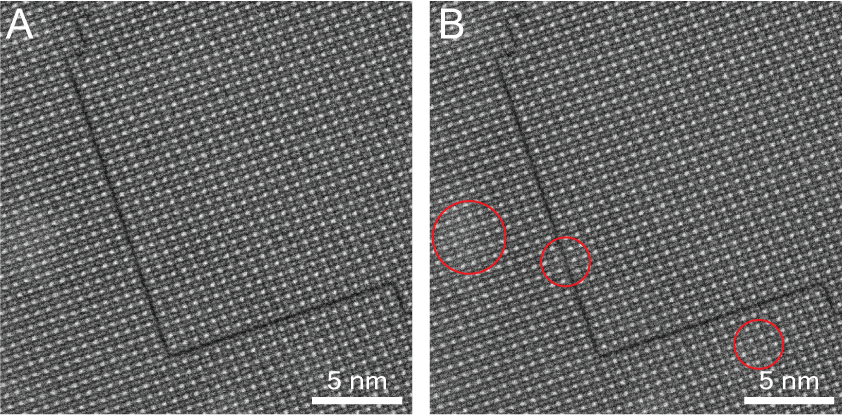


**Fig. S4. STEM-ADF images of a RP defect in γ-CsPbI_3_ films.** **(A)** Frame 1 (dose: 2.3 $\times$ 10^4^ e/A^2^) and **(B)** Frame 10 (accumulated dose: 2.3 $\times$ 10^5^ e/A^2^). Red circles highlight the higher intensity regions of the image arising from PbI_2_ clusters formed under the beam.

To investigate whether and at what dose the RP defect is modified under the electron beam, a series of 10 fast scan STEM-ADF images were collected from the same region of interest, each frame at a dose of 2.3 $\times$ 10^4^ e/A^2^. **Fig. S5** shows atomic-resolution STEM-ADF images acquired from the same region with two different electron doses. The RP defect is found to be extremely stable even under the total accumulated electron dose of 2.3 $\times$ 10^5^ e/A^2^. No obvious structural modifications were observed during the acquisition, as shown by the comparison of the first and the last frame in **Fig. S5**. 10 frames were then post aligned by a cross-correlation method, summed and ABSF (Average Background Subtraction Filter) filtered to further enhance the signal-to-noise ratio. All observations regarding the structure of the RP defect, including quantitative measurements of atomic positions and octahedral tilts, were checked and no difference could be detected between any of the images, irrespective of dose. From this we conclude that maintaining a dose below 2.3 $\times$ 10^5^ e/A^2^ will ensure the intrinsic RP planar structure is maintained. The dose used throughout this study was at least an order of magnitude lower than this.


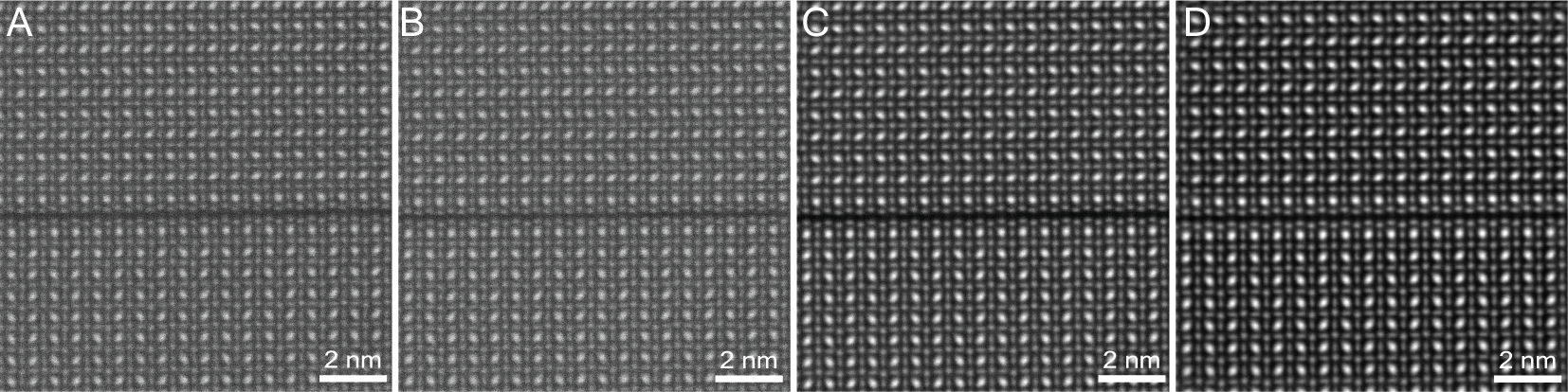


**Fig. S5. STEM-ADF images of a RP defect in γ-CsPbI_3_ films.** **(A)** Frame 1. **(B)** frame 10. **(C)** sum of 10 aligned image frames. **(D)** ABSF filtered image of **(C)**.

Supplementary Note 5 – 90° Crystal Rotation – [001]/[110] Axis Swap across RP gap

Fig. 2 reveals a 90°crystal rotation across the RP gap corresponding to a switch from the [001] to the [110] axis, as seen from the ellipticity analysis of Pb/I columns. This 90°crystal rotation can also be identified from the Fourier Transform (FT) of RP defect images, which show reflections from two crystal domains with orientations corresponding to the [001] and [110] axis, see for example the FT in Fig. S6C taken from the image in Fig. S6B. The corresponding images generated from the inverse FT using the blue/red circled reflections, Fig. S6D and Fig. S6E respectively, reveal the two different crystal domain orientations on either side of the RP gap. Specifically, it reveals the [001] directions in these two crystal domains are orthogonal, that is, there is a “swap” from the [001] to the [110] axis across the gap.


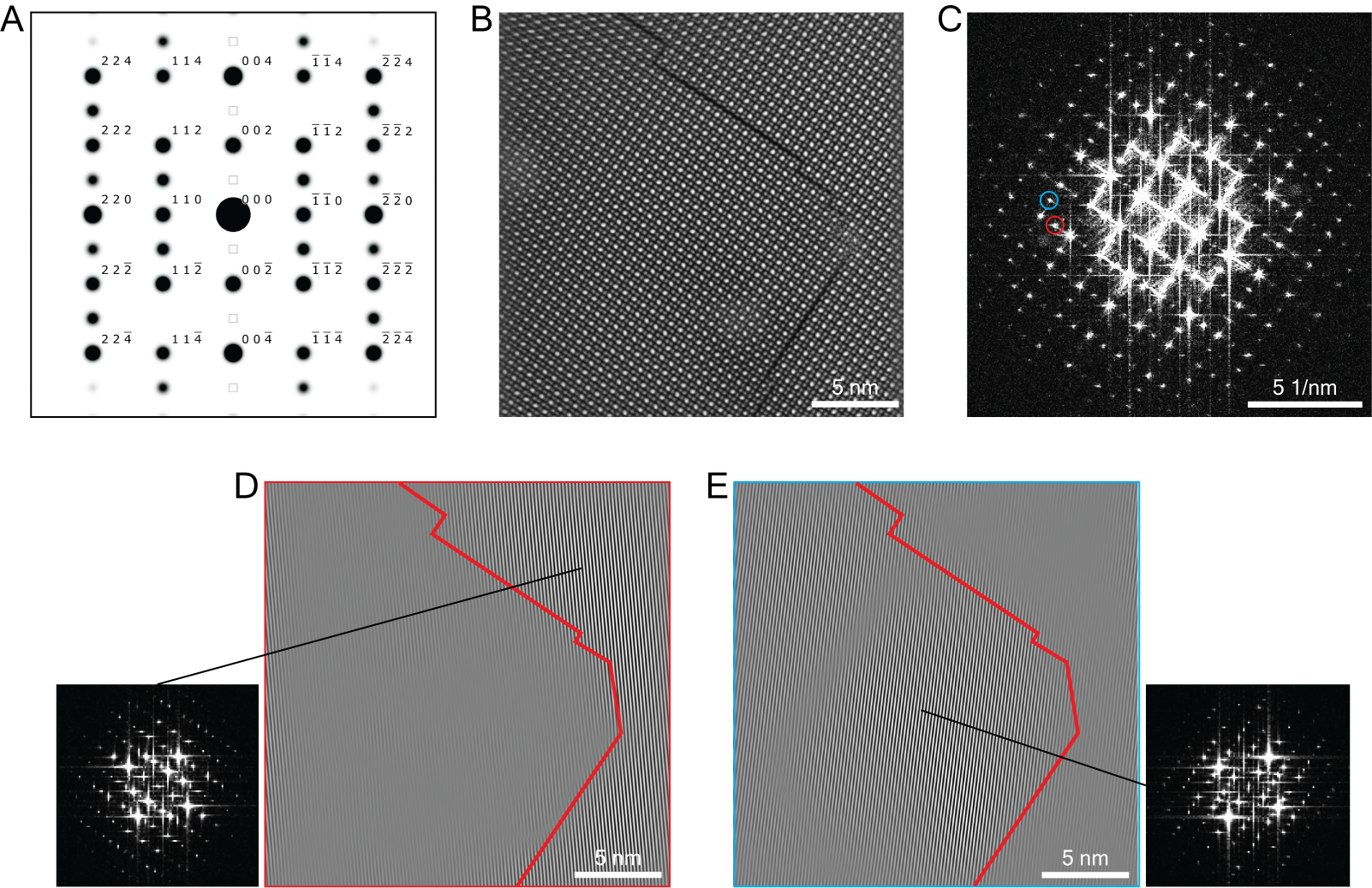


Fig. S6. 90°crystal rotation boundaries – [001]/[110] axis switch across RP gap (A) Simulated diffraction pattern of γ-CsPbI_3_ viewed in the [$\mathbf{1}\bar{\mathbf{1}}\mathbf{0}$] direction. (B) STEM-ADF image of a RP defect in Cs-1.1. (C) FT of the image in (B) with the 225 reflections circled. (D, E) Inverse FT image generated from the red and blue spots circled in (C), respectively.

A similar approach can also be applied to show the axis swap applies across the whole grain. Fig. S7 shows the same grain as in Fig. 2A (Note: Fig. 2A is rotated by 90° anti-clockwise to align the RP defect vertically for better visualisation). The 90°crystal rotation with orthogonal [001] axes across the RP defect is apparent from the inverse FT images shown in Fig S7 (B-C).


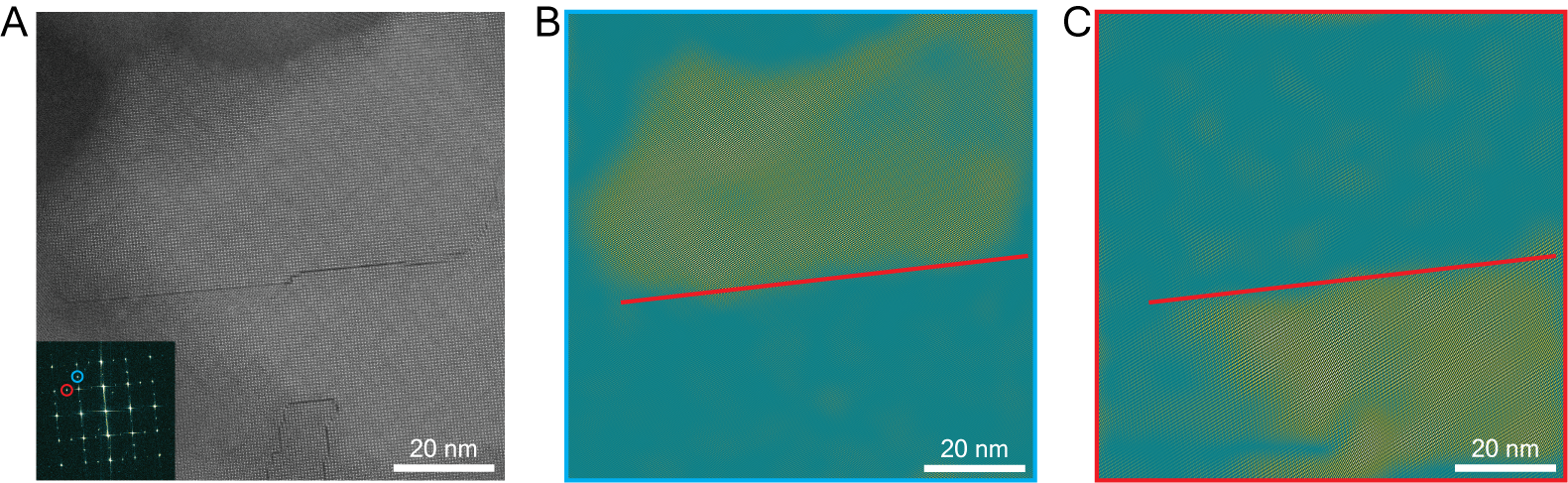


**Fig. S7. 90°crystal rotation boundaries – [001]/[110] axis switch across RP gap – Example 2 (A)** STEM-ADF image of a grain containing a RP defect in the Cs-1.1 film. **(B, C)** Inverse FT images using reflections from the two domains with orthogonal [001] directions, as circled by blue and red in **(A)**, respectively.

Supplementary Note 6 – Refinement of the Atomic Positions Associated with RP Planar Defects by STEM-ADF Simulations

Due to the nature of electron scattering within a crystal, the positions of the intensity maxima in a STEM image do not necessarily correlate exactly with the position of the corresponding atomic column in the specimen, particularly at an aperiodicity such as the RP defect. To quantify the atomic structure of the RP defects and provide a precise atomic model for DFT calculations later, we calculate STEM-ADF images of structural models using a multislice algorithm incorporating 30 frozen phonon configurations (MuSTEM ^[5]^) and refine the structural parameters until the positions of the intensity maxima in the calculated images match those measured in experiment.

There are many Inorganic Crystal Structure Database (ICSD) structure files for γ-CsPbI_3_ across which there are major differences in atomic positions and even space group. We therefore start by refining the ‘bulk’ γ-CsPbI_3_ crystal structure from our STEM-ADF images using one of the structure files (ICSD-21955) as our starting structure. We find the atomic positions of the Cs atoms to be identical to the ICSD-21955, but the octahedral tilt angle is smaller.

We then use this bulk structure for the periodic domain interiors and build an initial starting model for the RP plane by bringing two domains together separated by the gap, as appears nominally in the image.

We next performed STEM simulations of the Type-90 RP model for a variety of Cs positions and octahedral tilt angles to refine its structure. Comparisons between simulated STEM-ADF images and the experimental image lead to quantitative measurements of the change in octahedral tilt and Cs position relative to the periodic, defect-free crystal structure. The best fit to the experimental data was found with a 0.5 Å Cs displacement (i.e. 10% displacement in Fig. S8A) together with an octahedral tilt relaxation of 3.5° in the first layer adjacent to the RP gap (i.e. 25% and 50% for the first layer and interior layers respectively in reference to the ICSD-21955, Fig. S8B), while subsequent layers were unchanged from the bulk structure. These structural parameter refinements provide precise RP defect models as shown by the excellent match between simulated and experimental STEM-ADF images of the Type-90 RP model in Fig. S9. These refined RP defect models will be used for DFT calculations later in Fig. 7.


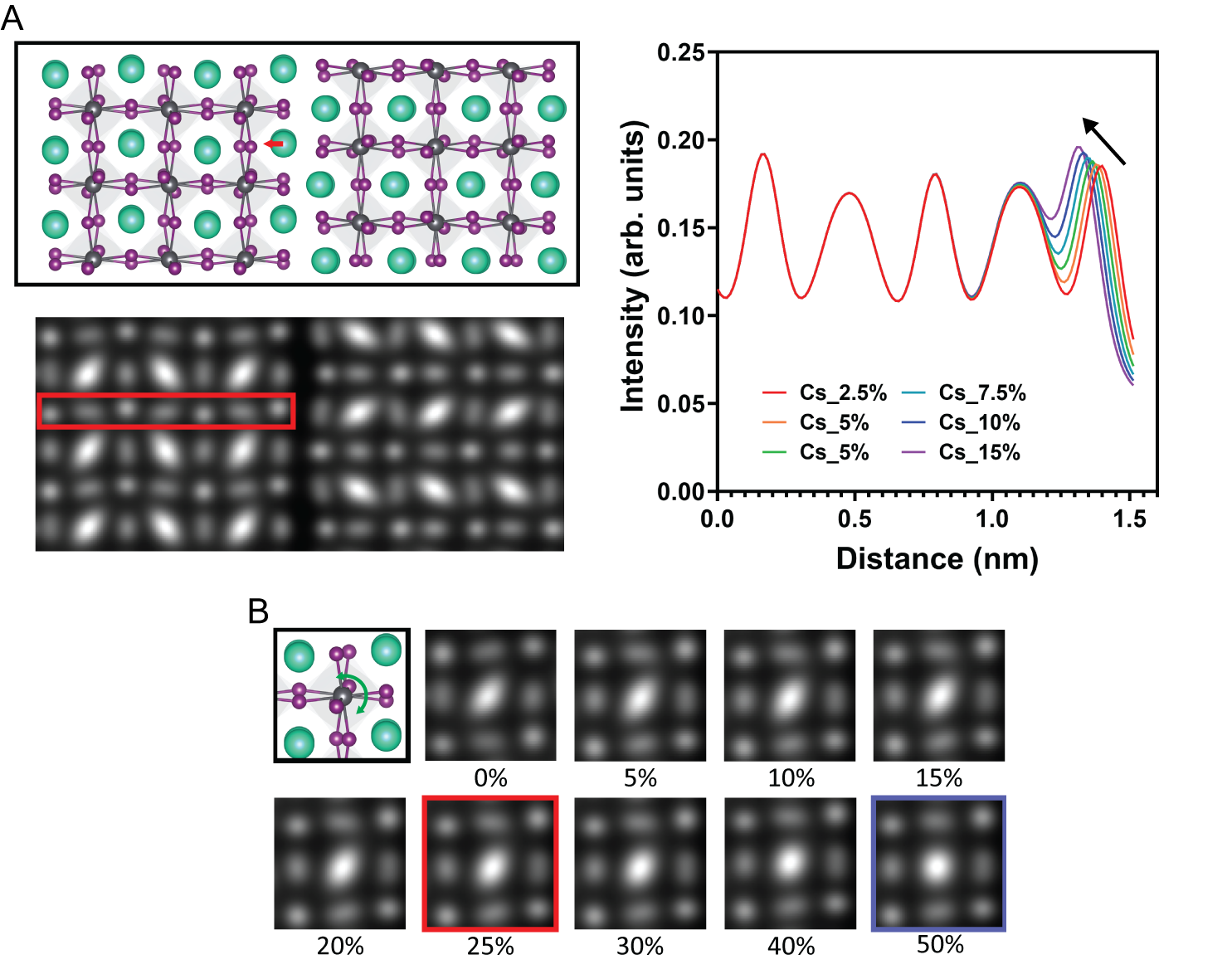


Fig. S8. STEM-ADF simulations on Type-90 RP models with varying Cs displacement distance and octahedral tilt relaxation. (A) The first layer of Cs atoms adjacent to the gap is displaced by 2.5%, 5%, 7.5%, 10%, 12.5% and 15% from the periodic position in the direction towards the domain interior (red arrow). (B) The octahedral tilt is relaxed by 0-50% in the direction indicated by the green arrow.


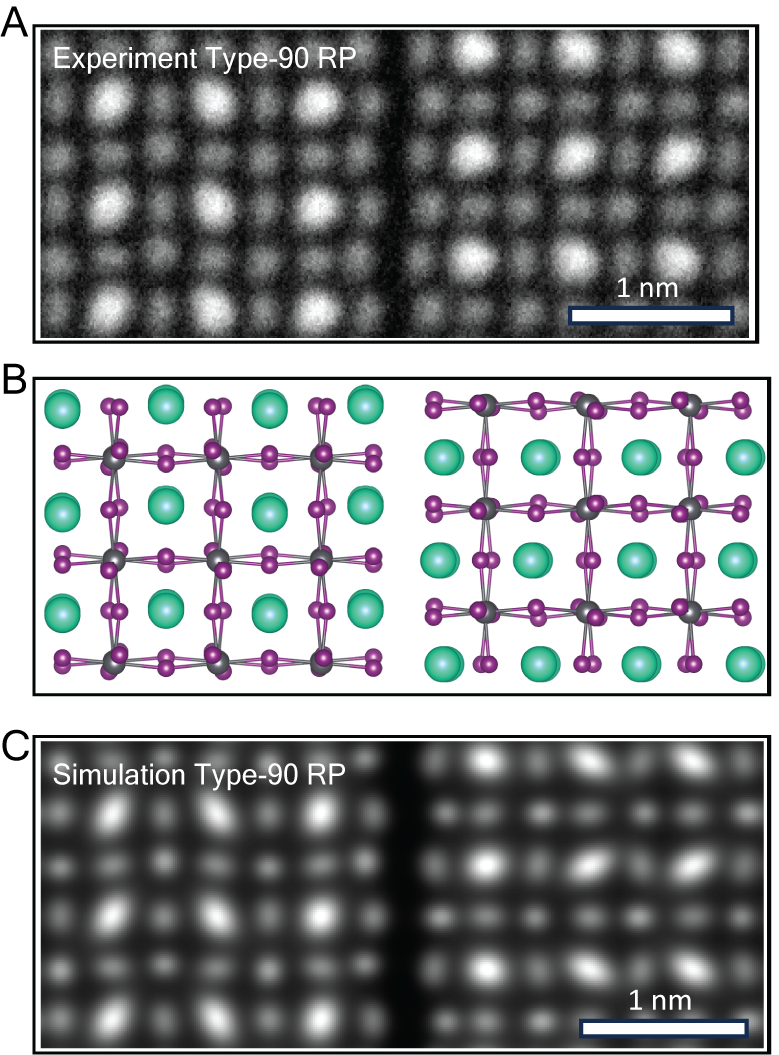


Fig. S9. Comparison between the refined Type-90 RP model and experimental data. (A) Experimental STEM-ADF image of a Type-90 RP planar defect. (B) parameter refined crystal model of Type-90 RP planar defect. (C) simulated STEM-ADF image of the refined model in (B).

Supplementary Note 7 – Type-0 RP Planar Defects

**Fig. S10** shows a representative Type-0 RP planar defect. In contrast to the Type-90 RP planar defect in **Fig. 2,** there is no crystal rotation of the domains each side of the RP gap, as illustrated by the ellipticity vector map in **Fig. S10B**. Except for the crystal rotation, the Type-0 RP planar defect is essentially the same as the Type-90 RP planar defect, comprising a displacement of the first Cs atom perpendicular to the gap (**Fig. S10C**) as well as a relaxation of octahedral tilt for the first layer (**Fig. S10B**). The alternating Cs displacement along the (110) RP plane is also consistent with Type-90 RP planar defects.


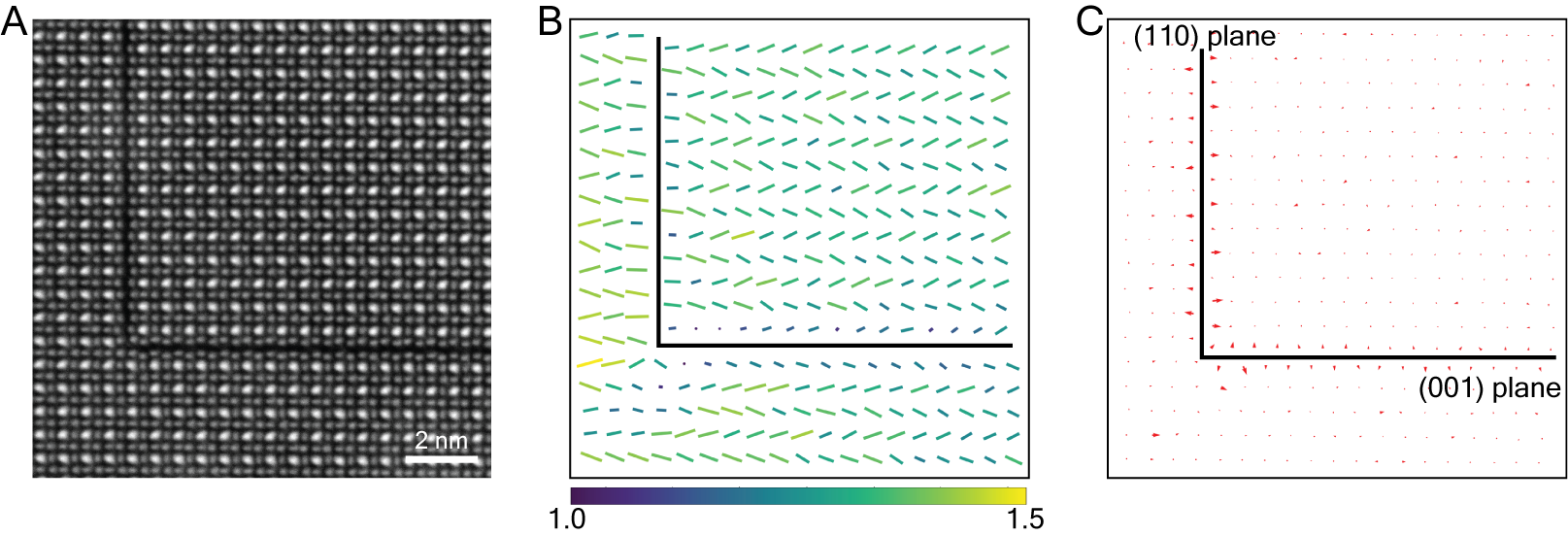


**Fig. S10.** **Quantitative analysis of the atomic structure at and around the Type-0 RP planar defect. (A)** Atomic-resolution STEM-ADF image of a representative Type-0 RP planar defect oriented in the [$1\bar{1}0$] zone axis in Cs-1.1 films. **(B)** Ellipticity vector map measured from Pb/I columns in **(A)**. Line direction indicates orientation of the ellipse major axis. Line length and colour indicates the magnitude of ellipticity (major axis / minor axis). **(C)** Displacement vector map of Cs columns measured from the Cs-Cs column distance in **(A)**.

**Supplementary Note 8** – **Measurements of the prevalence of RP defects**

Fig. S11 shows how the prevalence of RP planes and RP TPs (turning points) are defined and measured from STEM images. The specimen thickness is assumed to be the same across the field of view, so measurements of specimen volume, RP plane area and RP TP length reduce to measurements of specimen area, RP plane “distance” and RP TP number. Specimen area is defined by the image field of view and the distance of the RP planes and the number of turning points are directly measured from the image (red lines and blue circles, respectively). The prevalence of RP planes and RP TPs are then calculated based on images of 22 grains in Cs-1.0 specimens, 21 grains in Cs-1.1 specimens and 25 grains in Cs-1.25 specimens, respectively. Statistical results are summarised and shown in Fig. 5D.


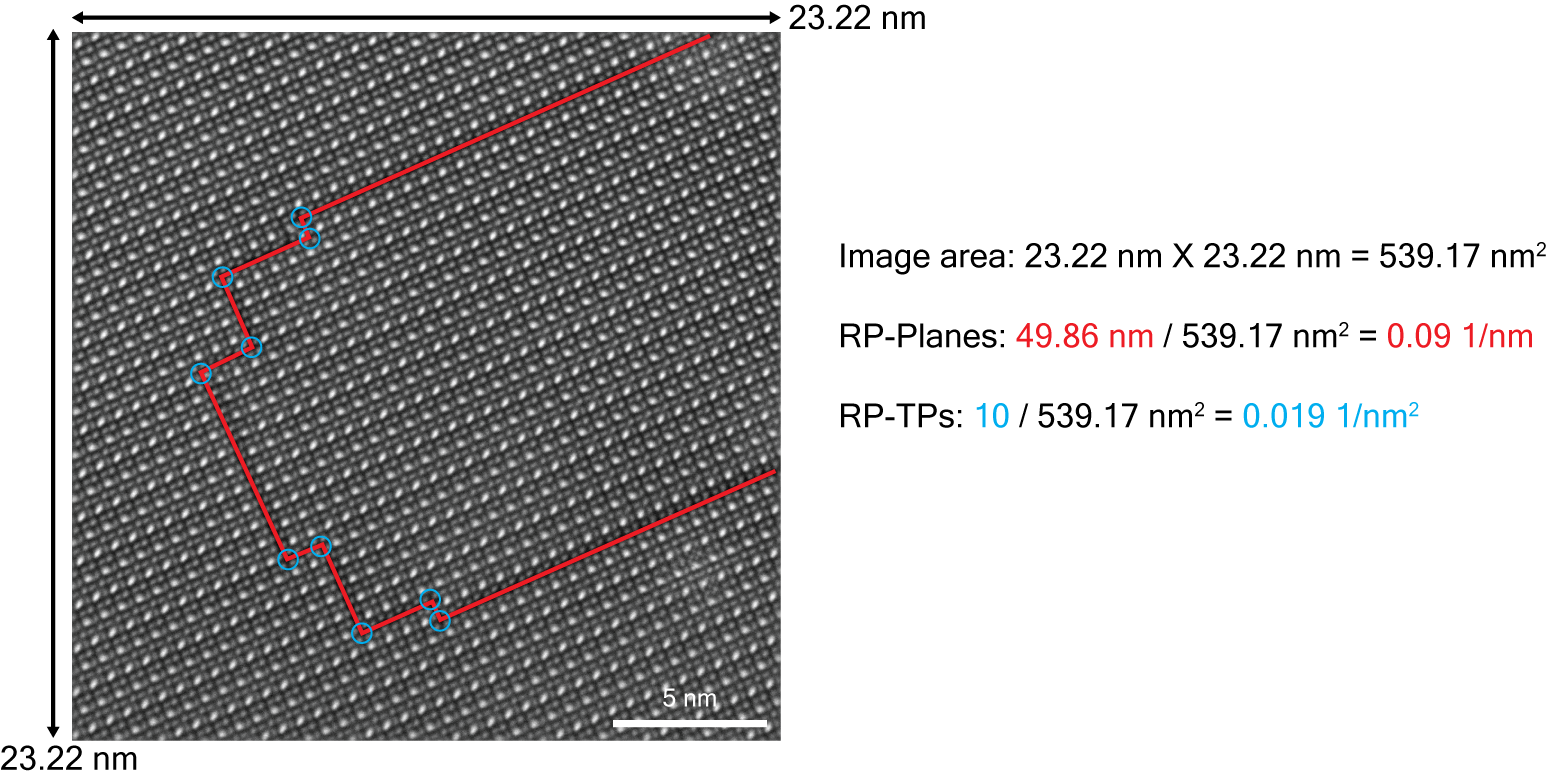


Fig. S11. Example of measurements of the prevalence of RP planes and RP turning points from a STEM-ADF image.

Supplementary Note 9 – Stoichiometric CsPbBr_3_ Films

CsPbBr_3_ films in its stoichiometric composition (Cs:Pb = 1:1) were prepared using the identical protocol as CsPbI_3_ films. Characterisation by TEM and XRD in Fig. S12(A-B) suggest the preparation of high quality polycrystalline γ-CsPbBr_3_ films. Stoichiometric CsPbBr_3_ films achieve a microstructure similar to that observed when 10% excess Cs is utilised to synthesise CsPbI_3_ (i.e. sample labelled as Cs-1.1). The difference in PL peak position in Fig. S12C shows the larger bandgap of CsPbBr_3_ compared with CsPbI_3_.


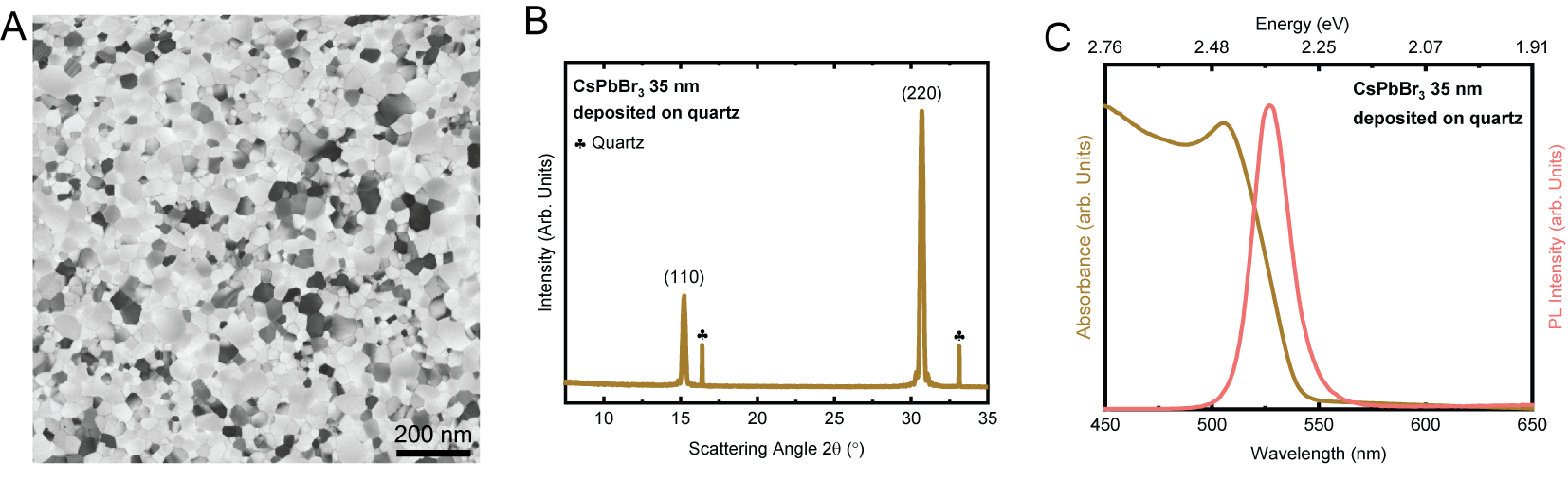


Fig. S12. Characterisation of 35 nm-thick stoichiometric CsPbBr_3_ thin films deposited on TEM grids or z-cut quartz substrates. (A) STEM-BF (bright field) image overview of the microstructure. (B) XRD pattern from a Cu-K_α_ 1.54 Å source. (C) Absorption and PL spectra. Thin films deposited on TEM grids and studied via STEM in this study were fabricated concurrently with films deposited on quartz. For obtaining the PL spectrum, a 398 nm-wavelength continuous wave laser was used to photoexcite the perovskite.

Supplementary Note 10 – 90° Rotation Domain Boundaries

Fig. 6(E-J) shows a gap-less (001)/(110) boundary in a CsPbBr_3_ film. The boundary and the corresponding 90° crystal rotation across the boundary can be identified from the ellipticity vector map measured from Pb/I columns, as shown in Fig. S13B. There are no measurable Cs displacements near this gap-less boundary (Fig. S13C), unlike the Type-90 RP defect in CsPbI_3_ which has a gap at the (001)/(110) boundary. This is consistent with the gap acting like a free surface, permitting the structure to relax with a Cs displacement and octahedral tilt relaxation.


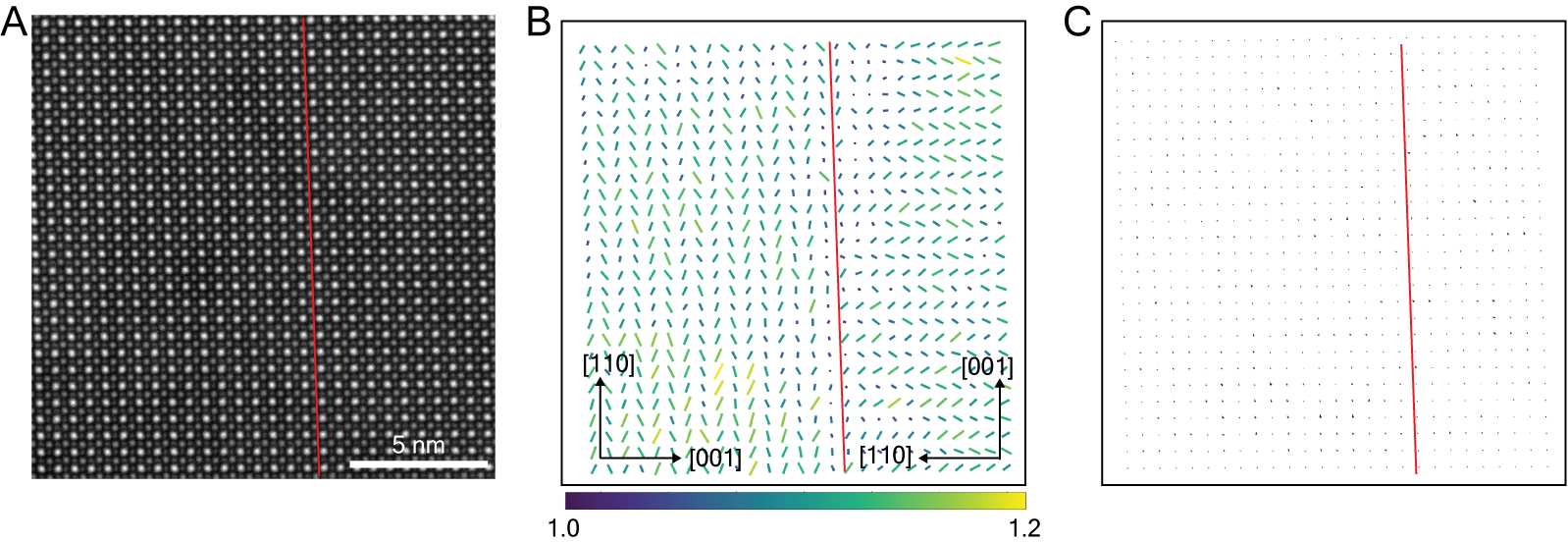


Fig. S13. Quantitative analyses of a gapless (001)/(110) domain boundary in a CsPbBr_3_ film. (A) STEM-ADF image of CsPbBr_3_ film oriented in the [$\boldsymbol{1}\bar{\boldsymbol{1}}\boldsymbol{0}$] zone axis showing a (001)/(110) domain boundary. Same image as in Fig. 6E. (B) Ellipticity vector map measured from Pb/I columns in (A) revealing the 90° crystal rotation across the boundary. (C) Cs displacement vector map measured from Cs-Cs column distances in (A), showing no Cs-displacement relative to the domain interior.

Supplementary Note 11 – Measurements of Photophysical Properties

**Fig. S14. Unnormalized photoluminescence spectra of an additional batch of 35 nm thick CsPbI_3_ films of different nominal Cs:Pb ratio deposited on quartz substrates.** Similar intensity trend to **Fig. 7A** is observed. For the sample with the nominal Cs:Pb ratio of 0.85:1, the sample degraded rapidly under photo-excitation. A 398 nm-wavelength continuous wave laser was used to photo-excite the perovskite.


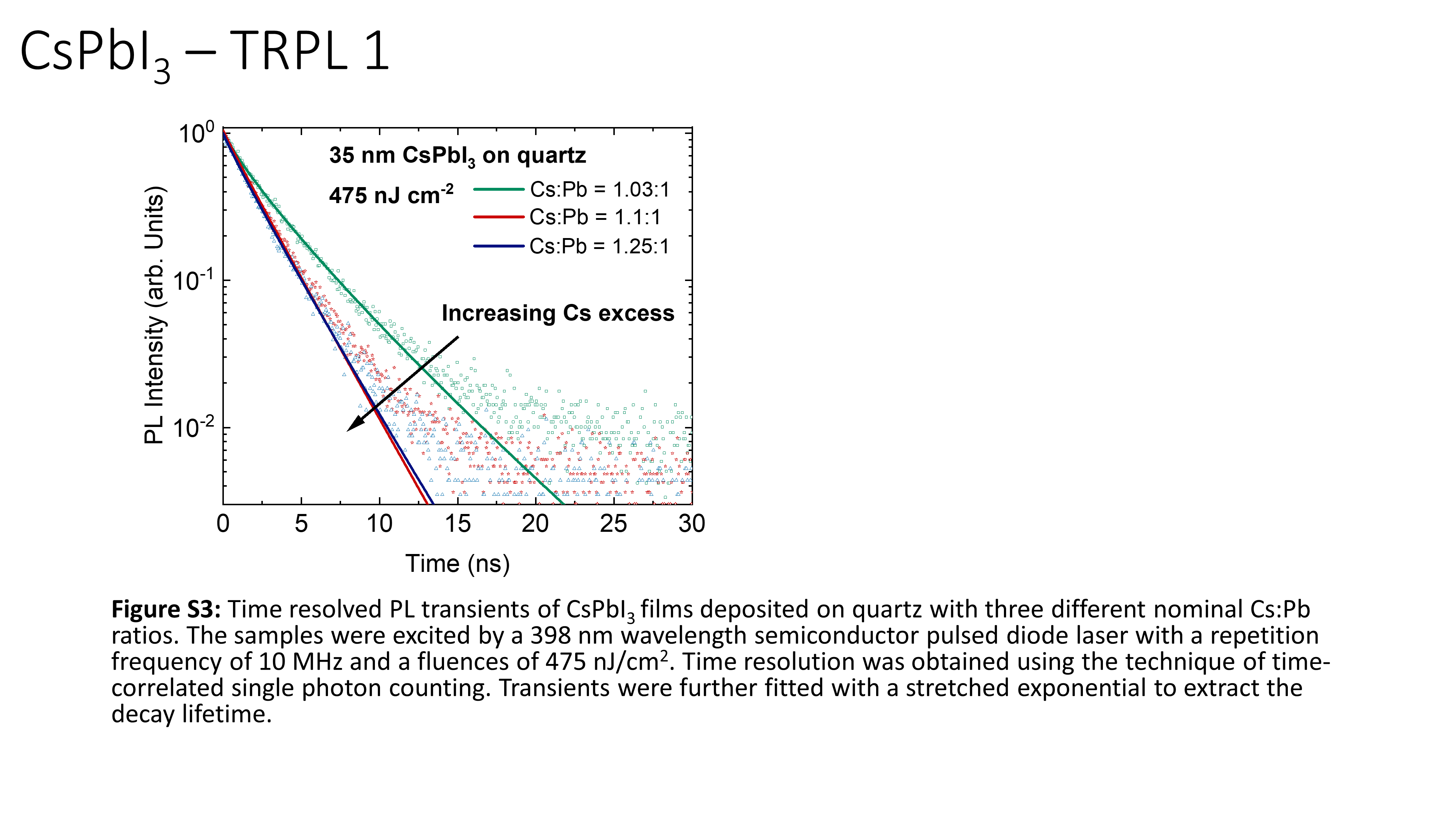


Fig. S15. Time resolved PL transients of CsPbI_3_ films deposited on quartz with three different nominal Cs:Pb ratios. The samples were excited by a 398 nm wavelength semiconductor pulsed diode laser with a repetition frequency of 10 MHz and a fluence of 475 nJ/cm^2^. Time resolution was obtained using the technique of time-correlated single photon counting. Transients were further fitted with a stretched exponential to extract the decay lifetime.


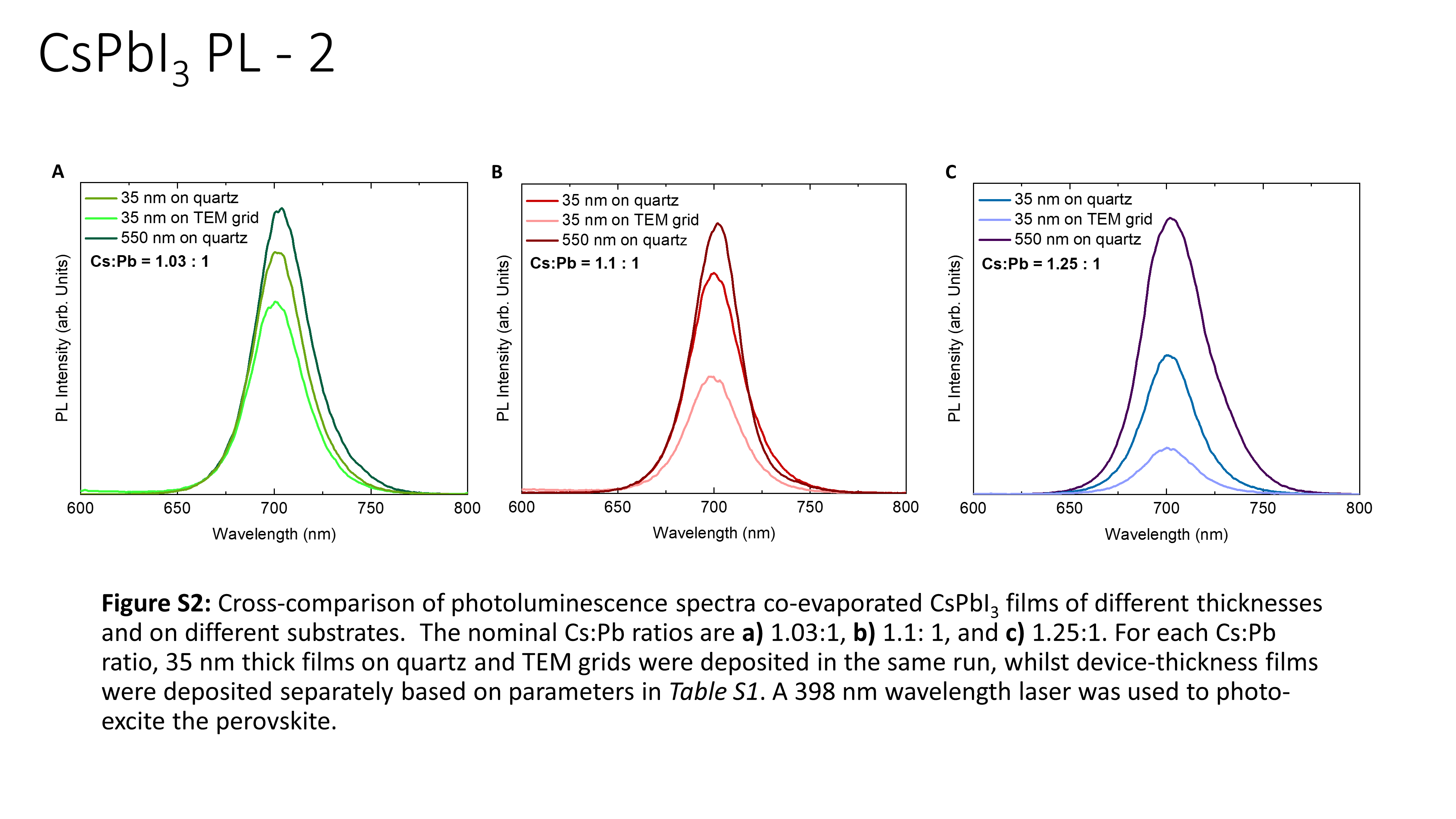


Fig. S16. Cross-comparison of photoluminescence spectra from co-evaporated CsPbI_3_ films of different thicknesses and on different substrates. The nominal Cs:Pb ratios are (A) 1.03:1, (B) 1.1: 1, and (C) 1.25:1. For each Cs:Pb ratio, 35 nm thick films on quartz and TEM grids were deposited in the same fabrication run, whilst device-thickness films were deposited separately based on parameters in Tab. S1. A 398 nm-wavelength continuous wave laser was used to photo-excite the perovskite.


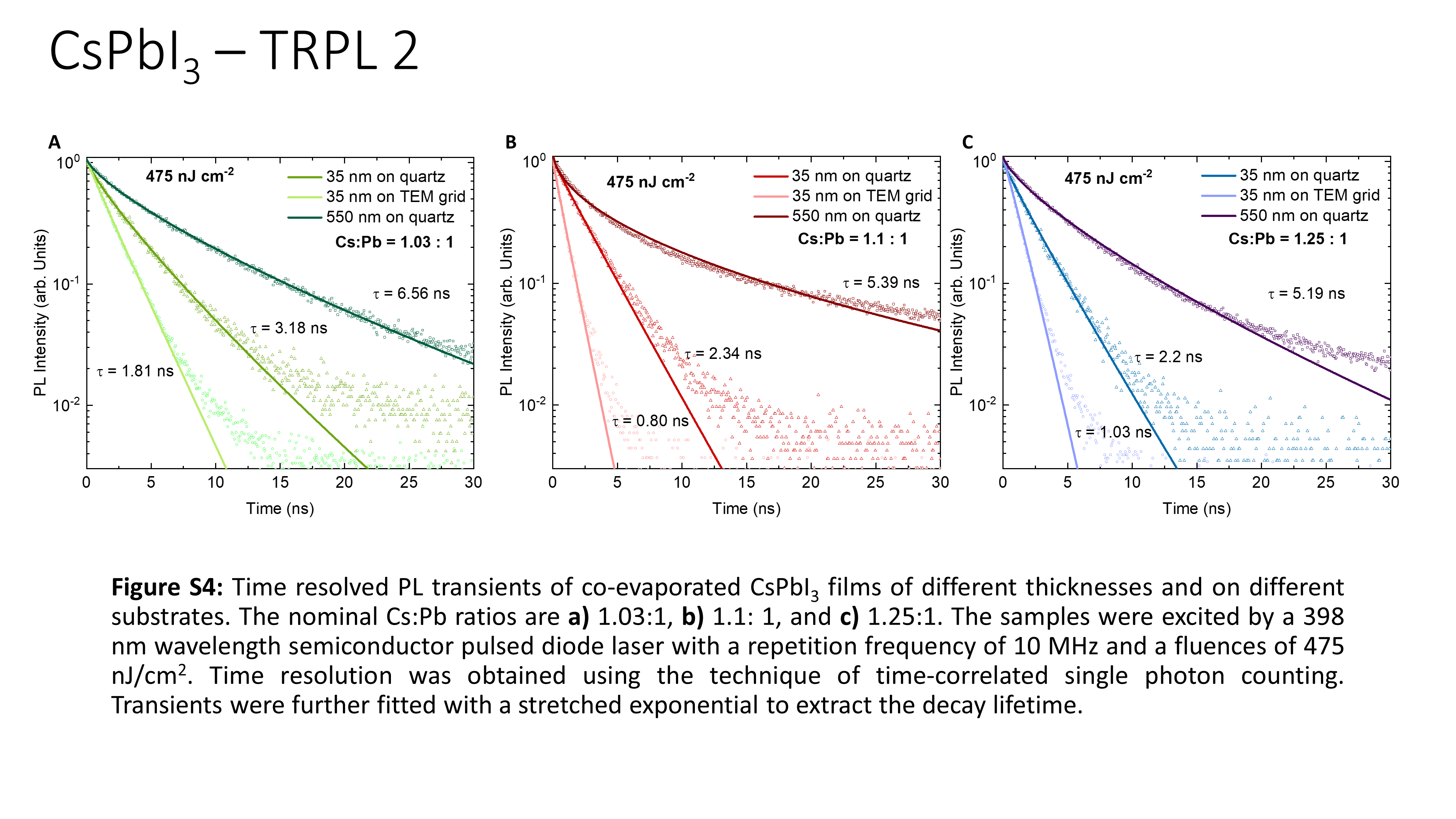


Fig. S17. Time resolved PL transients of co-evaporated CsPbI_3_ films of different thicknesses and on different substrates. The nominal Cs:Pb ratios are (A) 1.03:1, (B) 1.1: 1, and (C) 1.25:1. The samples were excited by a 398 nm wavelength semiconductor pulsed diode laser with a repetition frequency of 10 MHz and a fluences of 475 nJ/cm^2^. Time resolution was obtained using the technique of time-correlated single photon counting. Transients were further fitted with a stretched exponential to extract the decay lifetime.


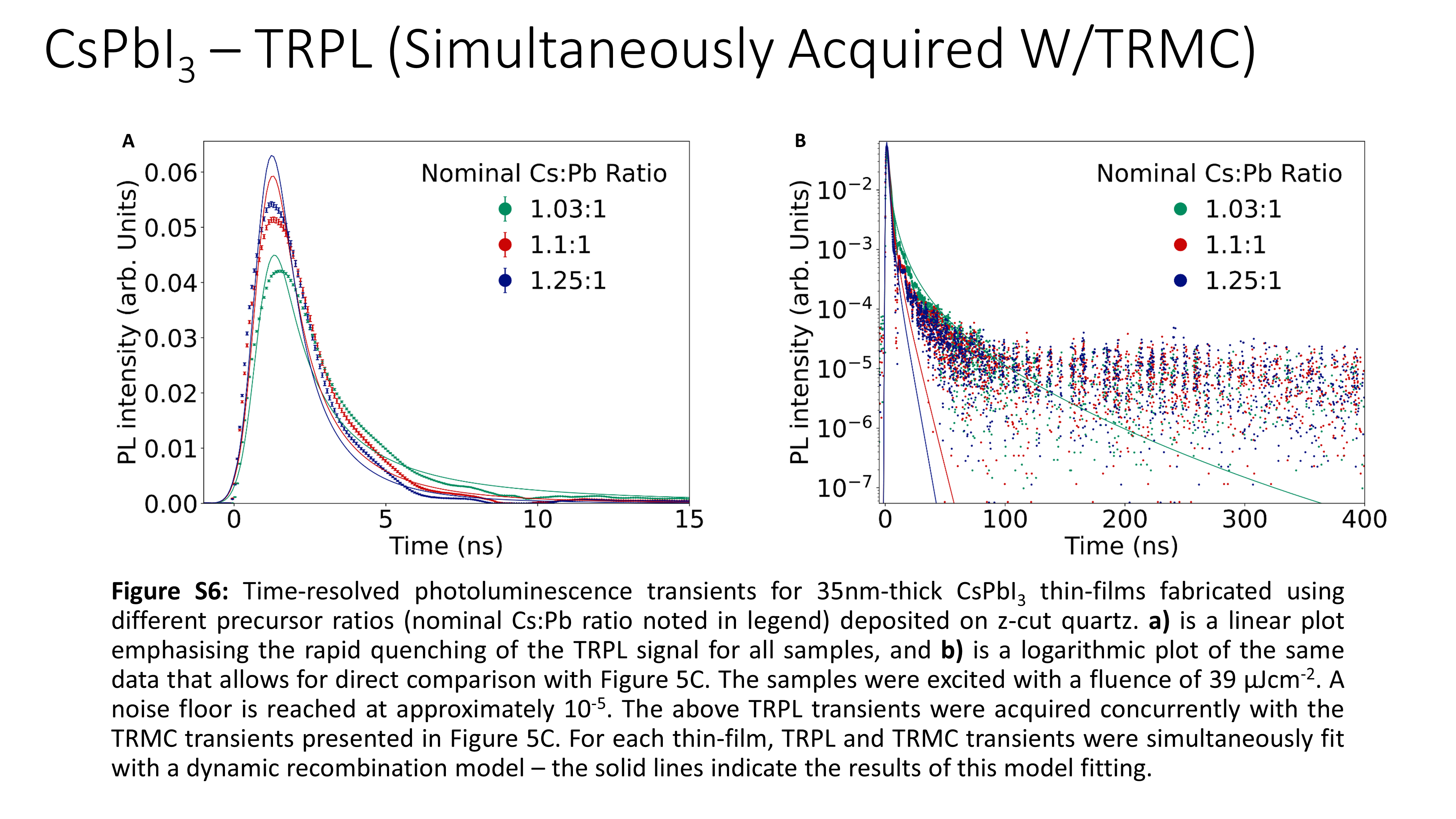


Fig. S18. Time-resolved photoluminescence transients for 35 nm-thick CsPbI_3_ thin-films fabricated using different precursor ratios (nominal Cs:Pb ratio noted in legend) deposited on z-cut quartz. (A) TRPL transients that were acquired concurrently with the TRMC responses presented in Fig. 7C, with (B) a logarithmic plot of the same TRPL data that allows for direct comparison with Fig. 7C. The samples were excited with a fluence of 32 μJcm^-2^. A noise floor is reached at approximately 10^-5^. TRPL and TRMC transients were simultaneously fit with a dynamic recombination model – the solid lines indicate the results of this model fitting.


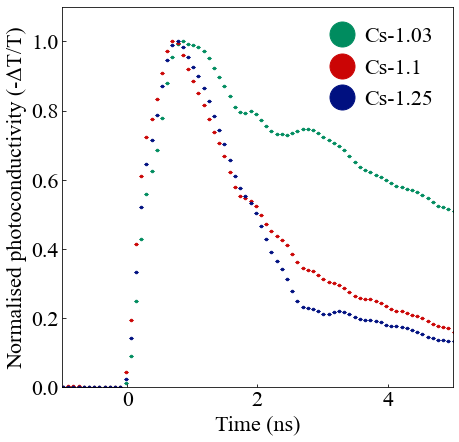


**Fig. S19:** Zoomed in TRMC transients from **Fig. 7C**, illustrating different changes of photoconductivity within the first few nanoseconds.

Supplementary Note 12 – DFT Structures Used for Computation

DFT calculations were performed on four structural models, as shown in Fig. S20.

All models stack two multi-layered slabs of CsPbI_3_ on either side of the defect and use for these slabs the unit cell parameters for the bulk crystal as refined from the STEM-ADF images above in Fig. S8 (Fig. S20A).

The first model, the “naïve RP model”, incorporates the additional Cs plane, the 3.5 Å gap and half-unit cell shift. However, it retains the same atomic positions at the RP plane, as occur in the bulk structure, which we know from our TEM measurements to be incorrect (Fig. S20B).

We then compare this naïve RP model with the correct Type-0 and Type-90 RP atomic structures measured from the TEM data which incorporate the Cs displacements and octahedral tilt relaxation, (Fig. S20C and Fig. S20D, respectively).


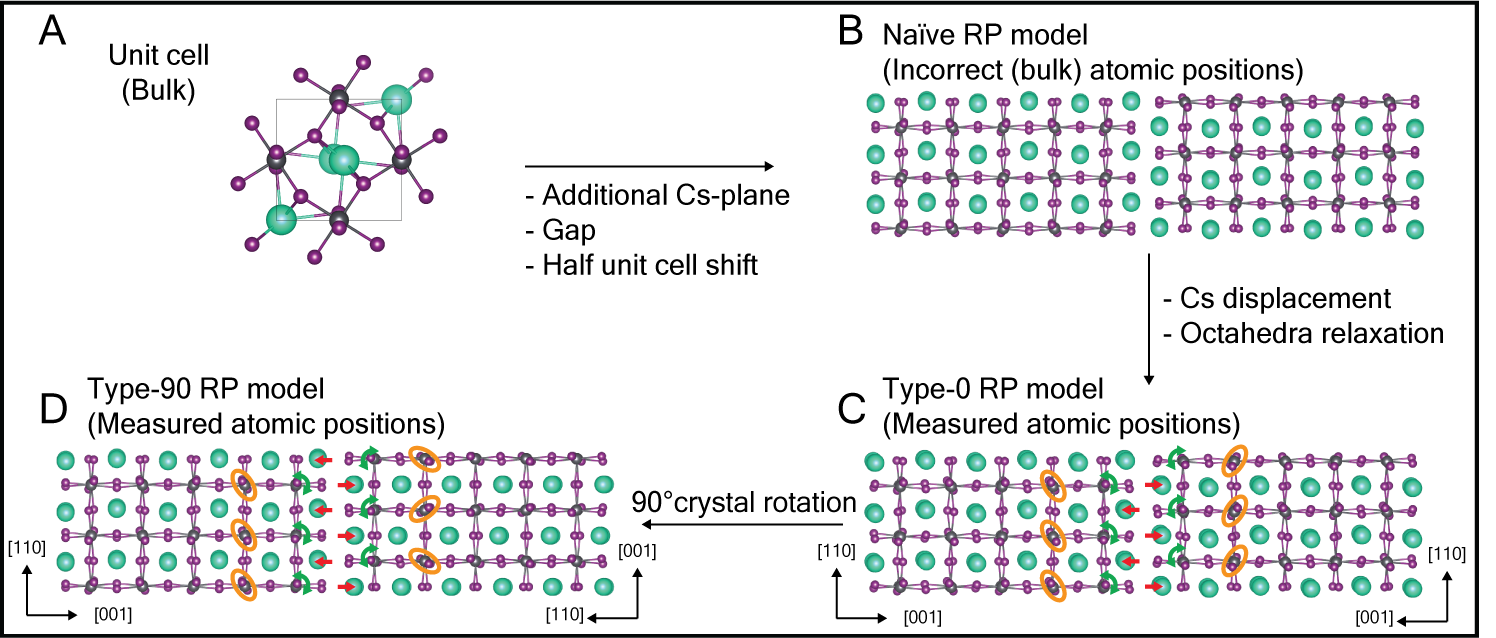


Fig. S20. RP models used in DFT calculations. (A) unit cell of γ-CsPbI_3_. (B) naïve RP model. (C) Type-0 RP model and (D) Type-90 RP model.

We note that, due to the constraints of periodic boundary conditions, the ’swapped’ lattice parallel to the defect interface was adjusted to accommodate the mismatch between the two slabs. Specifically, the lattice parameter was set to expand and contract respectively, with a maximum displacement of 0.58% for all the atoms in each slab. As a result, the two in-plane lattice parameters were set as 12.400 Å (modified from 12.472 Å and 12.328 Å for each slab) and 12.328 Å. For transparency, we include the structural models we use for our calculations as supplementary data.

The vacuum thickness between periodic replicas of the two slabs in the direction perpendicular to the RP defect was tested ranging from 3.5 Å, 13.5 Å to 23.5 Å, for both naive and correct models constructed from two 3 × 2 × 2 CsPbI_3_ supercell slabs. Based on the band gap dependence as shown in **Tab. S5**, a vacuum thickness of 13.5 Å was applied for all the systems throughout the study, to remove any interactions between periodic replicas of slabs pairs.

| High Symmetry Point | Type-90 model | | Naive model | Bulk structure |
| --- | --- | --- | --- | --- |
| Γ | 0.416 eV | 0.659 eV | | 0.345 eV |
| Χ | 0.416 eV | 0.659 eV | | 0.592 eV |

**Tab. S4.** Band gap values for the correct Type-90 RP model, the naïve RP model and the bulk orthorhombic CsPbI_3_ supercell at Γ and Χ point.

| Vacuum thickness | Type-90 model | Naive model |
| --- | --- | --- |
| 3.5 Å | 0.474 eV | 0.750 eV |
| 13.5 Å | 0.477 eV | 0.754 eV |
| 23.5 Å | 0.477 eV | 0.754 eV |

Tab. S5. Convergence of calculated band gaps with PBE + SOC on 3 different vacuum thicknesses for both the correct Type-90 RP model and naive model with a 3-layer slab.

**References**

[1] K. B. Lohmann, S. G. Motti, R. D. J. Oliver, A. J. Ramadan, H. C. Sansom, Q. Yuan, K. A. Elmestekawy, J. B. Patel, J. M. Ball, L. M. Herz, H. J. Snaith, M. B. Johnston, Solvent-Free Method for Defect Reduction and Improved Performance of p-i-n Vapor-Deposited Perovskite Solar Cells. *ACS Energy Lett.* **2022**, *7*, 1903.

[2] Q. Yuan, K. B. Lohmann, R. D. J. Oliver, A. J. Ramadan, S. Yan, J. M. Ball, M. G. Christoforo, N. K. Noel, H. J. Snaith, L. M. Herz, M. B. Johnston, Thermally Stable Perovskite Solar Cells by All-Vacuum Deposition. *ACS Appl. Mater. Interfaces* **2023**, *15*, 772.

[3] S. Yan, J. B. Patel, J. E. Lee, K. A. Elmestekawy, S. R. Ratnasingham, Q. Yuan, L. M. Herz, N. K. Noel, M. B. Johnston, A Templating Approach to Controlling the Growth of Coevaporated Halide Perovskites. *ACS Energy Lett.* **2023**, *8*, 4008.

[4] M. Nord, P. E. Vullum, I. MacLaren, T. Tybell, R. Holmestad, Atomap: a new software tool for the automated analysis of atomic resolution images using two-dimensional Gaussian fitting. *Adv. Struct. Chem. Imaging* **2017**, *3*, 1.

[5] L. J. Allen, A. J. D’Alfonso, S. D. Findlay, Modelling the inelastic scattering of fast electrons. *Ultramicroscopy* **2015**, *151*, 11.

[6] A. M. Ulatowski, K. A. Elmestekawy, J. B. Patel, N. K. Noel, S. Yan, H. Kraus, P. G. Huggard, M. B. Johnston, L. M. Herz, Contrasting Charge-Carrier Dynamics across Key Metal-Halide Perovskite Compositions through In Situ Simultaneous Probes. *Adv. Funct. Mater.* **2023**, *33*, 2305283.

[7] A. M. Ulatowski, A. D. Wright, B. Wenger, L. R. V. Buizza, S. G. Motti, H. J. Eggimann, K. J. Savill, J. Borchert, H. J. Snaith, M. B. Johnston, L. M. Herz, Charge-Carrier Trapping Dynamics in Bismuth-Doped Thin Films of MAPbBr3Perovskite. *J. Phys. Chem. Lett.* **2020**, *11*, 3681.

[8] C. Wehrenfennig, M. Liu, H. J. Snaith, M. B. Johnston, L. M. Herz, Charge-carrier dynamics in vapour-deposited films of the organolead halide perovskite CH_3_NH_3_PbI_3-x_Cl_x_. *Energy Environ. Sci.* **2014**, *7*, 2269.

[9] T. W. Crothers, R. L. Milot, J. B. Patel, E. S. Parrott, J. Schlipf, P. Müller-Buschbaum, M. B. Johnston, L. M. Herz, Photon Reabsorption Masks Intrinsic Bimolecular Charge-Carrier Recombination in CH_3_NH_3_PbI_3_ Perovskite. *Nano Lett.* **2017**, *17*, 5782.

[10] I. G. Hughes, T. P. A. Hase, Measurements and their Uncertainties: A practical guide to modern error analysis. *Measurements and Their Uncertainties: A Practical Guide to Modern Error Analysis*, Oxford University Press, Oxford, **2010**.

[11] L. M. Herz, Charge-Carrier Dynamics in Organic-Inorganic Metal Halide Perovskites. *Annu. Rev. Phys. Chem.* **2016**, *67*, 65.

[12] P. Hohenberg, W. Kohn, The convolution equation of Choquet and Deny on [IN]-groups. *Integr. Equations Oper. Theory* **1964**, *136*, B864.

[13] K. F. Garrity, J. W. Bennett, K. M. Rabe, D. Vanderbilt, Pseudopotentials for high-throughput DFT calculations. *Comput. Mater. Sci.* **2014**, *81*, 446.

[14] P. Giannozzi, O. Andreussi, T. Brumme, O. Bunau, M. B. Nardelli, M. Calandra, R. Car, C. Cavazzoni, D. Ceresoli, M. Cococcioni, others, Advanced capabilities for materials modelling with Quantum ESPRESSO. *J. Phys. Condens. Matter* **2017**, *29*, 465901.

[15] J. P. Perdew, A. Zunger, Self-interaction correction to density-functional approximations for many-electron systems. *Phys. Rev. B* **1981**, *23*, 5048.

[16] M. J. van Setten, M. Giantomassi, E. Bousquet, M. J. Verstraete, D. R. Hamann, X. Gonze, G. M. Rignanese, The PSEUDODOJO: Training and grading a 85 element optimized norm-conserving pseudopotential table. *Comput. Phys. Commun.* **2018**, *226*, 39.

[17] D. R. Hamann, Optimized norm-conserving Vanderbilt pseudopotentials. *Phys. Rev. B - Condens. Matter Mater. Phys.* **2013**, *88*, 085117.

[18] J. Even, L. Pedesseau, J. M. Jancu, C. Katan, Importance of spin-orbit coupling in hybrid organic/inorganic perovskites for photovoltaic applications. *J. Phys. Chem. Lett.* **2013**, *4*, 2999.

[19] M. U. Rothmann, J. S. Kim, J. Borchert, K. B. Lohmann, C. M. O. Leary, A. A. Sheader, L. Clark, H. J. Snaith, M. B. Johnston, P. D. Nellist, L. M. Herz, Atomic-scale microstructure of metal halide perovskite. *Science* **2020**, *370*, eabb5940.

[20] W. Li, M. Hao, A. Baktash, L. Wang, J. Etheridge, The role of ion migration, octahedral tilt, and the A-site cation on the instability of Cs_1-x_FA_x_PbI_3_. *Nat. Commun.* **2023**, *14*, 8523.

[21] S. Cai, J. Dai, Z. Shao, M. U. Rothmann, Y. Jia, C. Gao, M. Hao, S. Pang, P. Wang, S. P. Lau, K. Zhu, J. J. Berry, L. M. Herz, X. C. Zeng, Y. Zhou, Atomically Resolved Electrically Active Intragrain Interfaces in Perovskite Semiconductors. *J. Am. Chem. Soc.* **2022**, *144*, 1910.

[22] D. Zhang, Y. Zhu, L. Liu, X. Ying, C. E. Hsiung, R. Sougrat, K. Li, Y. Han, Atomic-resolution transmission electron microscopy of electron beam–sensitive crystalline materials. *Science* **2018**, *359*, 675.

[23] A. Alberti, C. Bongiorno, E. Smecca, I. Deretzis, A. La Magna, C. Spinella, Pb clustering and PbI_2_ nanofragmentation during methylammonium lead iodide perovskite degradation. *Nat. Commun.* **2019**, *10*, 2196.

[24] X. G. Zhou, C. Q. Yang, X. Sang, W. Li, L. Wang, Z. W. Yin, J. R. Han, Y. Li, X. Ke, Z. Y. Hu, Y. B. Cheng, G. Van Tendeloo, Probing the Electron Beam-Induced Structural Evolution of Halide Perovskite Thin Films by Scanning Transmission Electron Microscopy. *J. Phys. Chem. C* **2021**, *125*, 10786.
